# Negotiating Mortality: A Qualitative Systematic Review and Thematic Synthesis of the Psychosocial Experiences of Pancreatic Cancer Across the Illness Trajectory

**DOI:** 10.3390/cancers18193177

**Published:** 2026-10-01

**Authors:** Lana Cook, Gillian Prue, Caitlin McShane, Mei Krishnasamy, Kate Furness, Susan McLaughlin, Gary Mitchell

**Affiliations:** 1 School of Nursing and Midwifery, Queen’s University Belfast, Medical Biology Centre 97 Lisburn Road, Belfast BT9 7BL, UK; g.prue@qub.ac.uk (G.P.); cmcshane30@qub.ac.uk (C.M.); gary.mitchell@qub.ac.uk (G.M.); 2 Department of Nursing, School of Health Sciences, University of Melbourne, The Alan Gilbert Building, Level 6, 161 Barry Street, Carlton, VIC 3010, Australia; meik@unimelb.edu.au; 3 Department of Sport, Exercise and Nutrition Sciences, School of Allied Health, Human Services and Sport, La Trobe University, Bundoora, VIC 3086, Australia; kate.furness@latrobe.edu.au; 4 Northern Ireland Pancreatic Cancer (NIPANC), 384 Belmont Road, Belfast BT4 2NF, UK; director@nipanc.org

**Keywords:** pancreatic cancer, psychosocial, psycho-oncology, lived experience, living with and beyond cancer, coping and support needs, qualitative evidence synthesis, qualitative systematic review, supportive care

## Abstract

**Background/Objectives:** Pancreatic cancer is associated with poor survival and substantial psychosocial burden, yet little is known about the psychological, emotional, and social dimensions of patients’ experiences across the illness trajectory. This review aimed to identify, analyse, and synthesise empirical qualitative literature on the psychosocial aspects of receiving, living with, dying with, or surviving pancreatic cancer. **Methods:** Seven bibliographic databases were searched from inception to 4 September 2025, with reference list screening and forward citation tracking undertaken. Records were managed in Covidence. Following screening of 4357 titles and abstracts and 412 full texts, 30 papers representing 28 studies were included. Methodological quality was appraised using the JBI Critical Appraisal Checklist for Qualitative Research. Data were extracted into a shared Excel workbook and synthesised using Thomas and Harden’s thematic synthesis approach. **Results:** Four overarching themes were developed: (1) from ambiguous symptoms to the shock and threat of diagnosis; (2) navigating care, information, and support; (3) embodied disruption, relational change, and reworking everyday life; and (4) living on borrowed time and coping with uncertainty and mortality. Across the included studies, pancreatic cancer was experienced as a profound disruption to participants’ lives, futures, and sense of selves; involved complex informational and care-navigation work; altered everyday lives; and provoked ongoing uncertainty around progression, recurrence, and survival. **Conclusions:** Pancreatic cancer exerts a profound psychosocial impact across early and advanced disease, with psychological, social, and emotional challenges intertwined with complex symptomatology, functional decline, uncertainty, and mortality awareness. Thus, substantial unmet psycho-oncology and supportive care needs exist for patients and their families.

## Simple Summary

Pancreatic cancer is one of the deadliest cancers and many people do not live long after diagnosis. While much is known about physical symptoms, we understand less about what people go through emotionally, mentally, and socially. We wanted to learn more about this, so we reviewed studies exploring people’s experiences across different stages of the illness. We found that many people struggled to make sense of vague symptoms and experienced diagnosis as shocking and life-changing. People often faced challenges navigating healthcare services and accessing information and support. The disease and its treatment disrupted daily life, relationships, and roles at home or work. Many people lived with ongoing fears about cancer progression, recurrence, and mortality, relying on support from others and personal coping strategies. Our findings highlight substantial unmet supportive care needs and suggest improved care pathways with clearer, person-centred information and timely emotional, social, and psychological support.

## 1. Introduction

Despite improvements in survival and outcomes for several other cancers, progress in pancreatic cancer continues to be limited, partly due to a lack of advancement in screening and early detection compounded by the tumour biology itself [1,2,3]. Pancreatic ductal adenocarcinomas (PDAC) are the most common subtype, accounting for more than 90% of pancreatic cancers; these are characterised by aggressive local invasion and early metastatic spread [4], which, combined with a non-specific symptom profile, contributes to the late-stage diagnosis and poor prognosis associated with pancreatic cancer internationally. Most people are diagnosed at stage III or IV, when tumours are often unresectable due to vascular involvement and metastases; consequently, only a minority of people are eligible for potentially curative surgery [1,2,5]. Pancreatic cancer is widely recognised as a poor-prognosis disease (often called a ‘less survivable cancer’ [6]); the clinical realities of late diagnosis, limited treatment options, and a short and often uncertain prognosis mean that a sustained psychosocial impact is likely for those diagnosed. Yet, the psychosocial aspects of people’s experiences across the pancreatic cancer illness trajectory have not yet been comprehensively synthesised.

Psycho-oncology is an interdisciplinary field concerned with the psychological, emotional, social, and behavioural dimensions of cancer across the disease trajectory, including quality of life (QoL) and supportive and palliative care needs [7,8]. In the context of this field, ‘psychosocial’ refers to the interaction between the psychological and social factors that influence people’s ability to understand, navigate, and cope with the challenges of cancer, be that during diagnosis, treatment, survivorship, palliative, or end-of-life care. Accordingly, this review adopted a broad psychosocial outlook which encompasses the emotional, psychological, social, relational, existential, informational, and supportive care dimensions of people’s experiences. These domains are outlined in Table 1 and were informed by psycho-oncology literature and previous reviews of the lived, psychological, and social experiences of pancreatic cancer [9,10].

The existing literature suggests that pancreatic cancer is associated with significant psychological, emotional, and social challenges across the illness trajectory [11]; we know that compared with people with other cancers, people with pancreatic cancer experience worse psychological QoL and similar reductions in their physical and social QoL [12]. Worsening health-related QoL has been associated with declining cognition, depression, anxiety, distress, and reduced ability to cope with the disease; literature suggests this is due to awareness of the low curability and survival of pancreatic cancer [13,14] and ineffective assessment, management, and support for symptoms of pancreatic exocrine insufficiency [15,16,17,18], pain, and fatigue [14,19]. People often experience anger and depression in the initial, post-diagnosis period [14] and describe being driven to survive, but the transition to coping with their diagnosis and information needs relating to pancreatic cancer, treatment options, and associated symptoms can be overwhelming [20,21,22,23]. Subsequently, people with advanced-stage pancreatic cancer and those receiving treatment(s) for pancreatic cancer commonly experience high symptom burden [14,17,21,24,25,26,27,28]; changes to their identity, lifestyle, and roles; and a related reduction in their QoL [14,15,20,21,22]. Furthermore, as they adjust and learn to cope with the short-term outlook of pancreatic cancer and their associated hopes and fears around their future and ongoing survival, their information needs reflect these aspects of their journey [14,20,21,22,29]. Deepening our knowledge and understanding of people’s experiences is essential for the development of appropriate support interventions to meet patients’ psychosocial needs and enhance their psychological wellbeing.

Existing literature reviews in this area have predominantly synthesised quantitative evidence around quality of life, symptom burden, and treatment outcomes. Qualitative evidence has typically been included either as a minor component of mixed-methods reviews or in qualitative reviews focusing on specific aspects of pancreatic cancer, such as experiences of treatment, symptom management, or the supportive care needs of care partners. To our knowledge, there has been no previous systematic synthesis of qualitative evidence exploring people’s psychosocial experiences across the pancreatic cancer illness trajectory. By bringing together evidence from people at different stages of the journey—including diagnosis, treatment, adaptation to living with and beyond pancreatic cancer, advanced cancer, and end-of-life—this review aims to contribute a more nuanced and comprehensive understanding of the psychosocial challenges, coping processes, and supportive care needs associated with pancreatic cancer.

Although quantitative studies in this area offer important insights into symptom burden, quality of life, and psychological distress, these outcome measures provide limited insights into how individuals perceive, navigate, and adapt to the realities of pancreatic cancer. Qualitative research is well positioned to explore the meanings people attach to their lived experiences, and synthesising this evidence offers an opportunity to develop a richer understanding of these. Therefore, a dedicated qualitative synthesis was considered appropriate, since the aim was to understand, rather than quantify, how people experience, interpret, and adapt to the psychosocial challenges associated with pancreatic cancer across the illness trajectory, and to do this in depth.

In summary, this review aimed to synthesise empirical qualitative literature concerning the experiences, needs, and perceptions of people diagnosed with pancreatic cancer across the illness trajectory. To our knowledge, this is the first evidence synthesis to take this approach. The review addressed the following research question: What are the psychosocial aspects of people’s experiences when living with, dying with, or living beyond pancreatic cancer?

## 2. Materials and Methods

This qualitative systematic review was registered in PROSPERO (CRD42025635882) and is reported in accordance with PRISMA 2020 [30] and ENTREQ [31]; the PRISMA 2020 checklists can be found in Supplementary Materials, Table S1. Search terms were developed iteratively using a PEO framework, combining concepts relating to pancreatic cancer, qualitative research, and psychosocial experiences. The psychosocial search terms were informed by previous qualitative reviews and syntheses of the lived, psychological, and social experiences of pancreatic cancer [9,10].

Seven bibliographic databases were searched from inception to 04.09.2025: CINAHL Complete (EBSCO Information Services, Ipswich, MA, USA), Embase and MEDLINE (Ovid/Wolters Kluwer, New York, NY, USA), APA PsycInfo (American Psychological Association, Washington, DC, USA), Scopus (Elsevier, Amsterdam, The Netherlands), Web of Science (Clarivate, London, UK), and the World Health Organization Global Index Medicus (Geneva, Switzerland). We also screened the reference lists of relevant studies and reviews and undertook forward citation tracking using Google Scholar (Google, Mountain View, CA, USA), Web of Science, and Scopus. The full database-specific search terms are provided in Appendix A, Table A1, Table A2, Table A3, Table A4, Table A5, Table A6 and Table A7. The search history and results and the search strategies for each database are provided in Supplementary Materials, Tables S2–S9.

Records were imported into Covidence systematic review management software (Veritas Health Innovation, Melbourne, Australia; www.covidence.org) for screening. Title and abstract screening were undertaken by one reviewer (L.C.), with 10% of studies checked by a second reviewer (G.M.), and full-text screening was subsequently conducted independently by the same two reviewers. Any disagreements were resolved through discussion, with consultation from a third reviewer (G.P.) where necessary. Studies were considered if they focused on people with pancreatic cancer and explored the psychosocial aspects of their experiences across diagnosis, treatment, palliative care, or survivorship. Published empirical articles and successfully defended dissertations were eligible, as were mixed-methods studies with extractable qualitative data. Non-empirical literature, conference abstracts, reviews, and intervention studies were excluded. Full inclusion and exclusion criteria are presented in Table 1.

Methodological quality was assessed using the Joanna Briggs Institute (JBI) Critical Appraisal Checklist for Qualitative Research [32]. Two reviewers (L.C. and C.M.S.) independently appraised each study and resolved disagreements through discussion, with a third reviewer (G.P.) available to adjudicate unresolved disagreements. No studies were excluded based on appraisal outcomes; instead, appraisal findings informed interpretation of the review findings.

Data extraction was undertaken by one reviewer (L.C.) with supervisory oversight from G.M. using a structured extraction form developed for this review. Extracted data included study characteristics, setting, participant group, design and methods, and qualitative findings (descriptions and illustrative quotes) which aligned with the inclusion/exclusion criteria. All data were entered into a shared Excel workbook (Excel for Microsoft 365; Microsoft Corporation, Redmond, WA, USA), where entries could be reviewed for completeness, consistency, and accuracy. Extracted data informed the development of initial descriptive codes and the subsequent thematic synthesis. The coding process for each theme is illustrated in Appendix B, Table A8, Table A9, Table A10 and Table A11.

We conducted a thematic synthesis following Thomas and Harden’s [33] three-stage approach to identify and integrate themes across heterogeneous studies and to generate interpretative data beyond the findings of original authors.

Firstly, the first author (L.C.) applied line-by-line, inductive coding to study findings and illustrative quotes for each included paper in a Word document codebook (Microsoft Word for Microsoft 365; Microsoft Corporation, Redmond, WA, USA). Each meaning unit relevant to the review question was extracted and coded. Secondly, codes were grouped into descriptive categories, which were compared across studies and refined through supervisory (G.M.) discussion. Thirdly, we (L.C., G.M.) interpreted these categories further to develop higher-level analytical themes that encapsulated the psychosocial aspects of participants’ experiences of pancreatic cancer across the included studies.

## 3. Results

**Figure 1:**
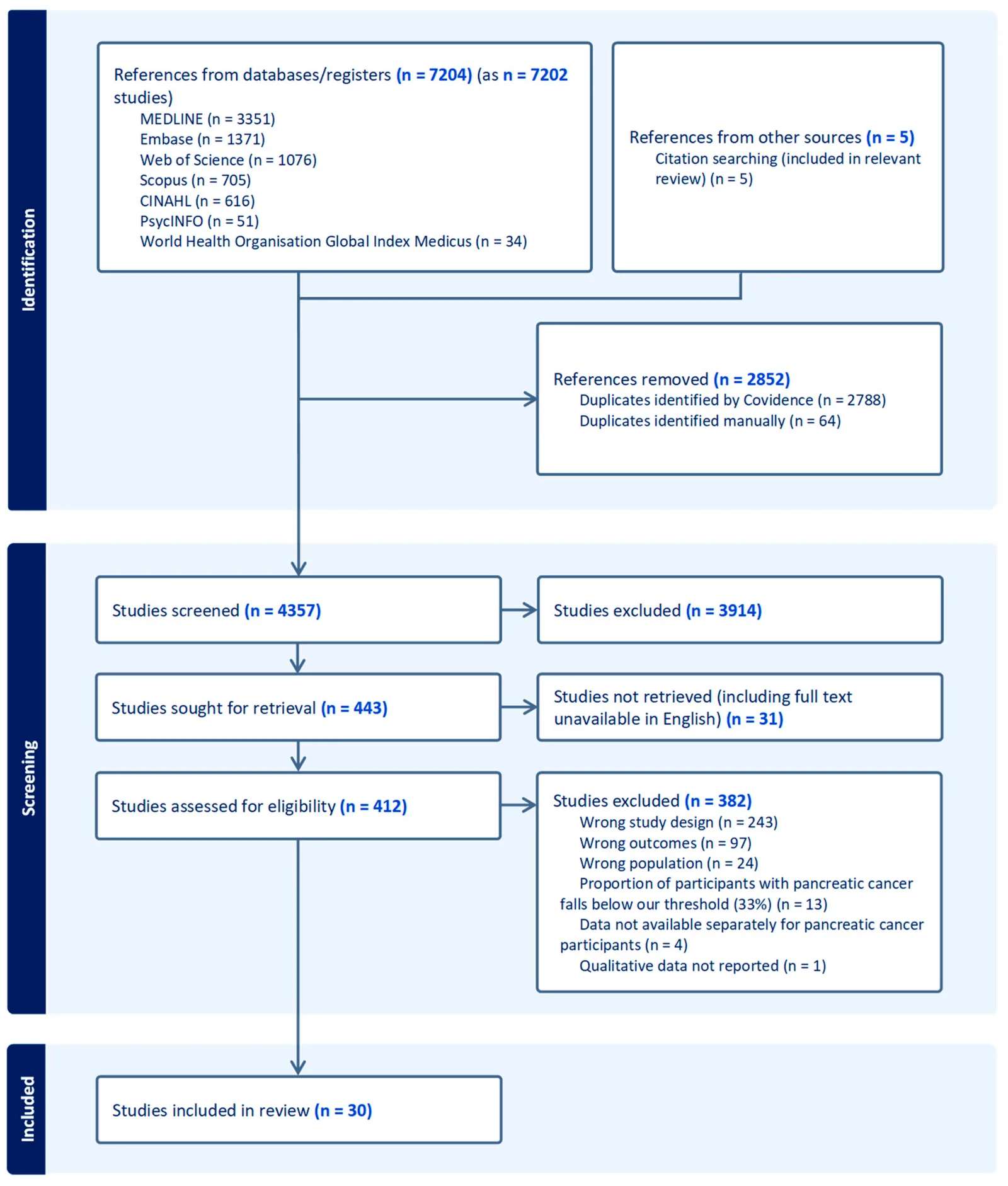
PRISMA flow chart.

Searches identified 7209 records (7204 from databases and five from citation searching). Of these, 2852 were duplicates (Figure 1), leaving 4357 for title and abstract screening. Following this, 3914 were excluded as irrelevant and 443 were sought for retrieval for full-text screening. Since full texts could not be retrieved for 31 studies, 412 studies were assessed for eligibility and 30 papers representing 28 studies were identified as eligible for inclusion: 29 qualitative papers (96.7%) and one mixed-methods paper with extractable qualitative data. Two of these papers [20,34] are from the same study, but each focuses on different aspects of their findings. Another two papers [35,36] likely report different aspects of the same dataset based on sample, setting, and authorship.

**Figure 2:**
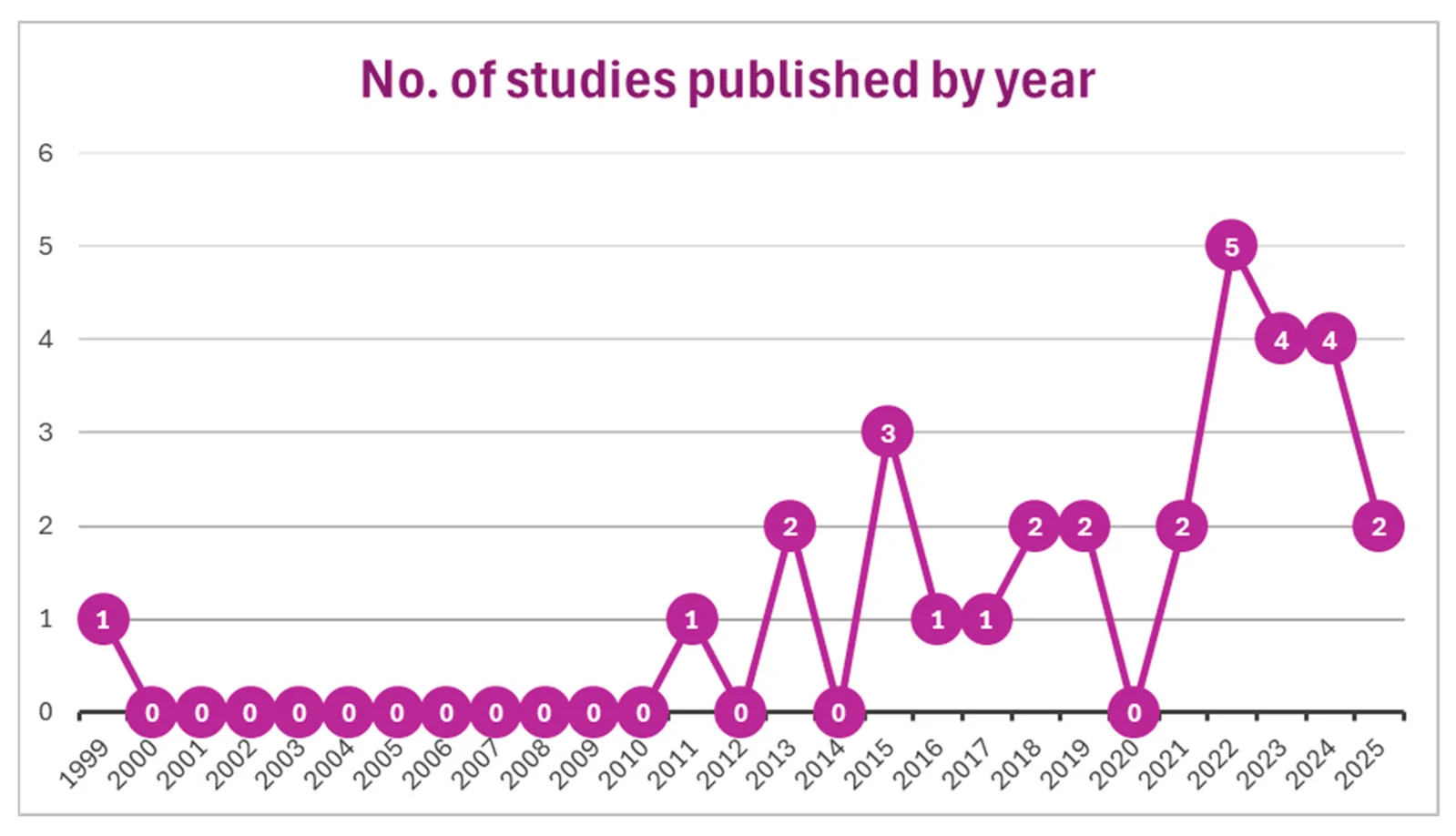
No. of studies published by year.

Papers were published between 1999 and 2025, with 26/30 (88.7%) of these published in the past decade (as per Figure 2), suggesting a significant increase in qualitative research exploring the experiences of people affected by pancreatic cancer. With the exception of two studies in China [37,38] and one in India [39], 90% of studies took place in high income countries (the USA *n* = 8 [14,21,26,40,41,42,43,44], the UK *n* = 7 [22,35,36,45,46,47,48]; Australia *n* = 3 [17,19,49]; Germany *n* = 3 [20,34,50]; China *n* = 2 [37,38]; and one each in Canada [29], India [39], Denmark [51], New Zealand [52], Greece [53], Israel [54], and The Netherlands [55].

All exclusion reasons are identified in Figure 1, but the top three reasons were wrong study design (*n* = 243), wrong outcomes (*n* = 97), and wrong population (*n* = 24).

Methodological quality appraisal was completed for all included studies using the JBI Critical Appraisal Checklist for Qualitative Research. Overall, study quality was variable, with common limitations relating to insufficient reporting of researcher positionality and reflexivity. No studies were excluded based on appraisal, but appraisal findings informed interpretation of the review findings.

Across the 30 included studies, data were collected from approximately 549 participants, including people diagnosed with pancreatic cancer, care partners and other family members (including bereaved relatives), and healthcare professionals. The total sample size was *n* = 600, including participants affected by other cancers. Most studies focused on people diagnosed with pancreatic cancer. Consistent with the epidemiology of pancreatic cancer, participants were predominantly older adults, including both men and women with mean or median ages ranging from their 60s–80s. However, the reporting of other participant characteristics was inconsistent and information on ethnicity, socioeconomic circumstances, disability, comorbidities, living arrangements, social support, and access to healthcare resources was often incomplete or absent. Much of the recruitment took place through specialist cancer services, follow-up clinics, or support networks, suggesting that the evidence base may disproportionately represent the experiences of people who were offered treatment and were able to access services and participate in research. Consequently, the experiences of people confronting social, economic, geographical, or health-related barriers remain less visible. Full study characteristics and the reporting of socioeconomic demographics across studies can be found in the Supplementary Materials, Tables S10 and S11, respectively.

The qualitative findings from the included studies were synthesised using an interpretive thematic approach guided by Thomas and Harden [33] to identify shared patterns in how individuals experience pancreatic cancer across the illness trajectory. Extracted qualitative data (participant quotations and author interpretations) were compared iteratively and descriptive categories were developed. Recurrent concepts were then grouped into broader analytical themes that captured common psychosocial experiences reported across the studies. These overarching, analytical themes move beyond descriptive findings of the primary papers to generate a higher-level synthesis of how symptom recognition, diagnosis, treatment navigation, the impact of symptoms and treatment on everyday life, and ongoing uncertainty were understood and lived by people diagnosed with pancreatic cancer and the people supporting them.

**Figure 3:**
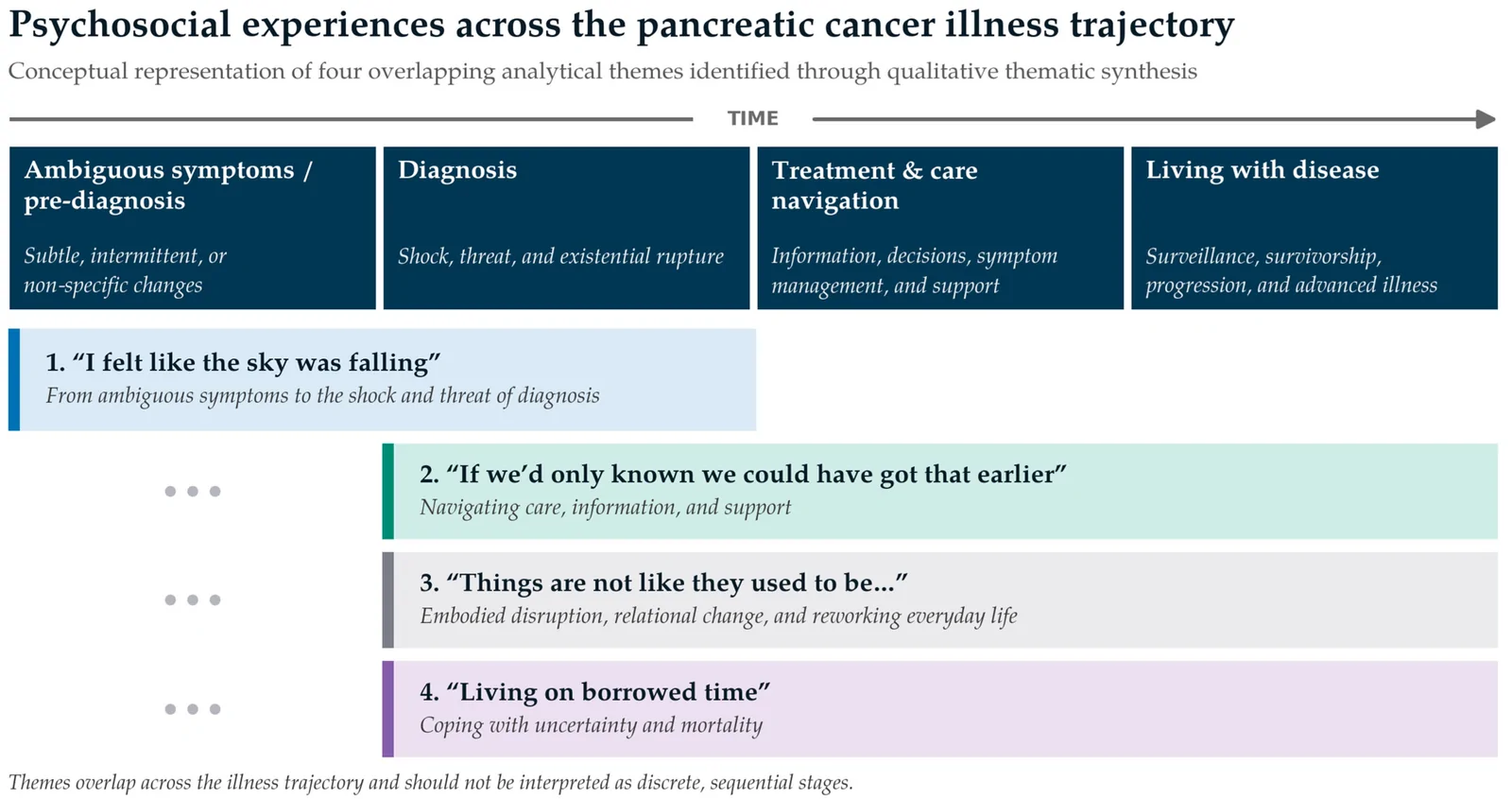
Conceptual representation of psychosocial experiences across the pancreatic cancer illness trajectory.

Four overarching themes were identified. The first theme, ‘“I felt like the sky was falling”: From ambiguous symptoms to the shock and threat of diagnosis’, explores how a diagnosis of pancreatic cancer transformed the interpretation of vague symptoms and was experienced as a profound disruption to participants’ lives and futures. The second theme, ‘“If we’d only known we could have got that earlier”: Navigating care, information, and support’, addresses how people with pancreatic cancer and their care partners navigated decision-making, information needs, accessed support, and coordinated complex care pathways. The third theme, ‘“Things are not like they used to be…”: Embodied disruption, relational change, and reworking everyday life’, considers how symptom burden, gastrointestinal changes, and treatment effects reshaped routines, relationships, social roles, identities, and family life, requiring ongoing adaptation to a changed everyday reality. The final theme, ‘“Living on borrowed time”: Coping with uncertainty and mortality’, describes how people attempted to keep living their lives despite persistent uncertainty, knowing that cancer recurrence, progression, and death remained possible and, for many, expected outcomes. Together, these themes illustrate how the bodily, relational, and existential dimensions of pancreatic cancer are experienced by people diagnosed with this cancer across the illness trajectory; the stages of the illness trajectory they encompass are presented in Figure 3.

### 3.1. Theme 1. “I felt like the sky was falling”: From ambiguous symptoms to the shock and threat of diagnosis

Across the included studies, pancreatic cancer was rarely experienced as emerging in a clear or orderly way. Rather, participants described an early phase in which bodily changes were subtle, intermittent, and easily normalised within everyday life [34]. Symptoms were attributed to diet, ageing, existing gastrointestinal problems, medication effects, or routine fluctuations in health, so the initial bodily disturbance often remained below the threshold of alarm:

“I remember going with my dad to the GP to find out answers [as to] why my dad had stomach aches … with this GP I had to go with him and say look can my dad have something done or can you forward this on…you have to push it along I think.”

[19]

In contrast, some people speak of bodily disturbances that did raise alarm, with one participant in Ristau et al. [34] stating, “I actually felt: something is seriously wrong with me.”

In Mills et al. [47], participants spoke of tiredness, appetite changes, vague abdominal sensations, and altered bowel function as things that “come and go,” making them difficult to interpret as evidence of serious disease. The diagnostic process analysed by Ristau et al. [34] similarly showed that the meaning and interpretations of symptoms were shaped by prior body awareness, earlier illness experience, and the pace at which symptoms accumulated, so that the seriousness of the situation often only became apparent retrospectively.

Where symptoms were present, they were often so vague and ambiguous that people were rarely prepared for the seriousness of a pancreatic cancer diagnosis. Since these bodily changes were interpreted as minor or benign, the point of diagnosis was experienced as a sudden rupture, disrupting ordinary life and making the future feel uncertain and threatened [14,22,34,35,38,47,49].

“There you are. Right in the middle of the week. Just like that. First, you could pull out trees, and now, now you have CANCER.”

[emphasis in original] [34]

“I was thinking, well, maybe it is just something that’s quite benign. You know, a benign growth. So when they did say it’s cancer that really… sledgehammered me… Cos I was still hanging onto this hope that it was benign, you know…”

[35]

“I don’t think I believed it because for something that I was told I had stones, which is quite a normal straightforward everyday occurrence, to having been told you have cancer was like two ends of the spectrum.”

[22]

When diagnosis was finally disclosed, it was repeatedly constructed as an event of overwhelming force. Participants across studies described being “hit… with a blow,” “run over by a train”, and “sledgehammered,” as they were confronted with a condition already culturally understood as nearly synonymous with death [34,35]. In Zhang et al. [38], a participant described, “When I first got my diagnosis, I felt like the sky was falling, my legs were shaking, my heart was panicking…” and in Ristau et al. [34], one participant recalled,

“That was actually my worst moment, when he told me that [I had pancreatic cancer]. And I couldn’t do anything with it yet. I didn’t even know yet/only had in the back of my mind: ‘Okay, you’ll die from this within a very short time.’”

In Bleicher et al. [40], participants used equally stark language, including “I thought I was doomed… that was the death knell”, “you hear that, and it’s like okay, here’s your death certificate” and “this was basically a death sentence.” The same immediate association between pancreatic cancer and mortality appeared in other studies [14,21,35,38,39], where the diagnosis generated fear, bodily panic, disbelief, and anticipatory grief not only for patients but also for spouses and relatives. The shock was intensified by the way that many participants understood themselves before diagnosis: as healthy, active, disciplined people who had lived responsibly. In Gibson et al. [49], participants explicitly contrasted the cancer diagnosis with an identity built around health, self-management, and physical competence: “…I did all of that healthy stuff, so good friends, good food, no excesses, so why do you get cancer? I don’t know.” While Wong et al. [14] showed how anger could emerge from the perceived mismatch between personal conduct and disease onset, questioning why this had happened to someone who didn’t have any of the risk factors:

“The ‘why me’ came on really strong…I couldn’t shake that. This has been thrown at me. I did nothing to deserve it…I never smoked, I don’t drink, I was health conscious, I’m not diabetic. I went through all of the risk factors and had none of them.”

In this sense, diagnosis was not merely bad news; it was an assault on people’s identity and assumptions about the future [14,34,35,38,39,40,49,52]. This existential shock also compressed time. Once a pancreatic cancer diagnosis was given, participants often felt they moved abruptly from uncertainty to an emergency state. In Taylor et al. [35], treatment unfolded with frightening speed:

“It all happened very quickly, all out of the blue really. Totally unexpected… I saw the specialist within a week [of the blood tests investigating anaemia], and she operated the next week… It was very, very quick; it was… [quiet laugh] what’s happened here? I only came for a blood test.”

In some studies [20,21,41,50], participants framed the early treatment phase as being less a period of careful deliberation than a necessary response to an urgent threat. One participant in Schildmann et al. [50] stated they were told, “You must do this, otherwise you won’t live to see the next half year,” and participants in Wancata et al. [21] similarly reflected, “I kind of feel like I didn’t have a choice”; “…because I think doing nothing is, that wasn’t even an option, to just let it be…” Although treatments were presented as options by clinicians, many participants felt that they weren’t making a genuine choice. The poor prognosis associated with pancreatic cancer meant that patients felt that declining surgery or treatment offered to them was unimaginable, as ‘doing nothing’ was perceived as allowing the cancer to progress unchecked and leaving little hope of extending survival. In fact, some participants suggested that they would rather die during surgery than not proceed with it, because death was otherwise felt to be inevitable [35]. For those eligible or potentially eligible for resection, the desire to “get to surgery” therefore functioned as more than a treatment goal; it represented access to continued life, however uncertain or burdensome that might be [20,21,35,40,41,50]. This finding should be interpreted in the context of the relatively small proportion of people diagnosed with pancreatic cancer who are eligible for surgery, despite these individuals being well represented in the available literature. As a result, the experiences of people with unresectable disease or poorer functional status may be under-represented in the evidence base and therefore less visible in this review and synthesis.

At the same time, this early phase was not emotionally uniform. Although fear dominated in the literature, participants similarly described denial, disbelief, and attempts to contain the implications of diagnosis. Bleicher et al. [40] described depression, acceptance, denial, positive thinking, and fighting attitudes within participants’ accounts, highlighting the dynamic and evolving approaches people took to understand and cope with their diagnosis over time.

“Yes, well, that might be such a bad diagnosis. But the tumor chose the wrong one. And there must be some who survive this whole thing. Otherwise, the small numbers don’t come about… ‘This guy [the cancer] picked the wrong guy. And that asshole is out.’”

[34]

Additionally, Zhang et al. [38] described “denial and silence” among both patients and spouses, particularly where families sought to protect one another from intolerable information. Fitzsimmons et al. [22] likewise identified coping strategies such as blocking knowledge and defending against distress. These responses should not be understood simply as maladaptive reactions. Rather, they appear to reflect the extremity of pancreatic cancer as a diagnostic event and the limited capacity people may have to process information and its implications when confronted with what they perceive to be a threat to their lives.

This interpretation aligns with the grounded theory developed by Ristau et al. [20], which proposes that the phase immediately following diagnosis is characterised by an acute period of survival, during which not dying becomes the dominant organising concern around which people orient their thoughts, choices, and actions. Seen across the studies, the early illness experience is therefore marked by a paradox: before diagnosis, symptoms were often too ambiguous to command attention; after diagnosis, the meaning of those symptoms became so abruptly clear that many participants felt that their futures had suddenly become uncertain, threatened, and likely significantly shortened [20,22,34,38,40,47]. In summary, depression, fear, denial, positive thinking, and fighting attitudes were not contradictory responses but different ways of coping with a diagnosis that was experienced as an acute threat to people’s survival.

### 3.2. Theme 2. “If we’d only known we could have got that earlier”: Navigating care, information, and support

Another finding across the studies is that decision-making was rarely experienced as a single bounded event. Instead, participants described a shifting process in which trust in clinicians, the search for information, and the practical work of navigating treatment were constantly renegotiated. In the immediate aftermath of diagnosis, many patients preferred a relatively delegated model of decision-making. In Schildmann et al. [50], patients explicitly prioritised trust over autonomous scrutiny, with one participant stating, “One also needs a bit of trust in the doctor or total trust in such a thing.” Bleicher et al. [40] similarly found that many participants were satisfied with a more passive role if they believed the surgeon was guiding them toward the treatment with the best chance of cure. In Stevens et al. [41] and Wancata et al. [21], neoadjuvant therapy or multimodality treatment was often experienced as “the only option,” while Taylor et al. [35] showed that the rapidity of treatment decisions could leave little emotional space for detailed reflection. Rather than indicating disinterest, this pattern suggests that in the acute phase of a life-threatening diagnosis, delegating decisions may function as a pragmatic response to fear, urgency, and limited cognitive capacity [20,21,35,40,41,50].

However, the apparent simplicity of this initial trust often gave way to patients and families needing to work hard to find information, ask questions, and keep track of what was happening in their care. Once the immediate crisis was underway, patients and caregivers had to assemble meaning across multiple appointments, professionals, treatment stages, and symptoms. Griffioen et al. [55] is especially important here in showing that decision-making continued throughout the disease journey, encompassing not only chemotherapy or surgery but transport, nutrition, family arrangements, and trial participation too. Their data make visible the volume of otherwise unrecognised “work” involved in coordinating cancer care. Fleszar-Pavlovic et al. [44], Khan et al. [19], Landers et al. [52], and Taylor et al. [36] all show that patients and families frequently sought information from the internet, other patients, relatives working in health care, or repeated contact with professionals because information delivered at diagnosis was either too much, too little, or poorly timed. The issue was therefore not just the amount of information but its sequencing, clarity, and relevance to the patient’s immediate concerns. As one participant in Fleszar-Pavlovic et al. [44] explained, information was manageable when “given little by little as the process was going.” By contrast, Khan et al. [19] document how dietary advice could become bewildering, with one participant saying, “probably the worst thing was finding out what I should be eating,” and turning to “Mr Google” in the absence of clear guidance [19,36,44,52,55].

This burden of sourcing and making sense of information was especially evident in relation to nutrition, enzyme replacement, surveillance, and symptom monitoring. Pancreatic enzyme replacement therapy (PERT) is used to treat pancreatic exocrine insufficiency (PEI), a condition in which inadequate activity and/or production of pancreatic enzymes means that food is not being digested properly, impacting on the absorption of nutrients [56]. PEI is commonly experienced by people with pancreatic cancer and manifests in symptoms such as weight loss, steatorrhoea, abdominal pain, and diarrhoea; administration of PERT aims to replace these enzymes, thereby improving nutritional status and addressing symptoms related to PEI [57].

In Dunleavy et al. [48] and Gooden and White [17], participants described receiving PERT without adequate explanation, leaving them unclear about what it was for, how to use it, and whom to ask when symptoms persisted. Participants in Dunleavy et al. [48] stated they were told to take PERT with “no explanation offered whatsoever”, “(t)hey just give it to you and because you don’t know any better, you just do what they say.” Some participants also lacked knowledge of the effects of undergoing a pancreatic resection, which organs and glands were involved in their procedures, and symptoms to expect afterwards as a result. Fleszar-Pavlovic et al. [44] note a participant experienced “a significant consequence resulting from the unintended use of an oil-based pain reliever”, not having been informed that their gallbladder was removed during their Whipple procedure. Additionally, Dunleavy et al. [48] found that many of their participants believed their “characteristic symptoms of malabsorption” were due to taking PERT itself, as they were not aware that PERT seeks to redress the pancreatic enzyme insufficiency caused by their cancer and/or resection:

“I am just putting it all down to Creon myself now because it has got to be that! I wasn’t like this before I started taking the Creon. It has to be causing the wind and the sickness.”

[48]

In their grounded theory, Ristau et al. [20] summarise the information and support needs around managing the impact of post-surgical complications and symptoms such as pain, inability to eat, intractable diabetes, and significant digestive issues:

“Teaching how to take pancreatic enzymes and manage pancreatic diabetes is prone to error in many hospitals due to a lack of experience and knowledge of the medical and nursing staff…Generally, in this acute situation, people with pancreatic cancer are forced to face their health problems. They react by gathering information…while they are also forced to endure the symptoms and side effects and feel correspondingly helpless.”

In contrast, Wancata et al. [21] found participants were able to reflect on conversations with the healthcare team about surgery andwhat organs it would involve, “including hand-drawn diagrams”. Furthermore, some describe recovering with “minimal complications”, although others felt that the difficulty of their recovery was “magnitudes past what they had expected” [21]. Beyond surgery, Fleszar-Pavlovic et al. [44] found that participants had been given information and understood it, but often “struggled with how to apply it, especially regarding chemotherapy side effects…leading to feelings of surprise and unpreparedness when symptoms occurred”:

“They didn’t explain it was the neuropathy. That is the major thing you get, so that has to be put in the forefront, not like a maybe you’ll get it. Everybody that gets that type of Folfirinox will get neuropathy. It’s the extent of neuropathy you have to be careful with.”

As a result, they recommended “more actionable and relevant guidance”, particularly in relation to the “extensive but impractical” nutrition guidance received [44].

Similar problems described by Ristau et al. [20] appear in Landers et al. [52], Khan et al. [19], Milliron et al. [42], and Taylor et al. [36], where food, digestion, weight loss, and medication management became domains in which patients and caregivers were expected to self-manage under uncertain and sometimes contradictory advice. In other situations, supportive care and the discovery of interventions and therapies such as PERT came only after a period of potentially avoidable distress, difficult symptoms, uncertainty, and/or persistent help-seeking, with bereaved care partners in Gooden and White [17] acknowledging the “huge difference” PERT made for their loved ones, but suggesting that these opportunities for symptom relief had been identified late in the illness trajectory:

“If we’d only known we could have got that earlier. That would have been great”.

“We only got it [pancreatic enzyme supplement] about a week or 10 days out [before she died] and it would have been better if we’d had them a month earlier.”

Lack of clear, timely information had a significant impact on how patients and their families navigated everyday aspects of care; they shaped eating behaviour, confidence, distress, and willingness to seek help, and they often transferred responsibility from specialist services onto patients and caregivers without adequate support [17,19,36,42,48,52], with the burden of educating GPs about aspects of their care, including PERT, falling to patients themselves [17,36].

“We brought up with him ‘pancreatic enzymes’. He [clinician] was-Oh, I don’t know whether that’s a good idea. We explained a few things, and he seemed very reluctant to give them.”

[17]

Surveillance and follow-up generated a distinct but related form of uncertainty; test results could provide reassurance while simultaneously provoking fear of cancer recurrence and progression [29,35,41,43,51]. In Dengsø et al. [51], testing CA-19-9 levels became a powerful but ambiguous biomarker through which hope and dread were repeatedly recalibrated. Some patients wanted results immediately and organised their emotional world around them; others withdrew from testing because the threat of hearing information they did not want to receive about their prognosis had become intolerable, with participants in Dengsø et al. [51] stating “I stopped because it just became too much so I simply stopped” and “if I’d said no all along I would have been better off and not had all those negative thoughts in my head all the time”. Another stressed:

“…every time I’ve consulted a nurse or a physician, I feel… I must be ready to say STOP [to an increased CA-19-9 or other information indicating recurrence]. Don’t say anything… I don’t want you to say anything when I am here alone… Don’t tell me anything…”

Whereas Deobald et al. [29] found that follow-up was valued both because it reassured patients and because it preserved the possibility of acting quickly if recurrence emerged. These studies indicate that information is never neutral in pancreatic cancer. It may reassure, overwhelm, compel vigilance, or force confrontation with recurrence risk, depending on when it is delivered, how it is explained, and what emotional resources patients and caregivers have available to receive it [29,35,43,51].

Running through all of this was ongoing uncertainty around who was responsible for different aspects of care and patients and families often found themselves unclear on who to approach or contact at different points of the illness trajectory [19,36,37,55]:

“When I told them that I was taking Creon and that it made me constipated… they didn’t care… it was stupid to even mention it… and maybe they (health care provider) aren’t even the ones responsible for doing anything about it.”

[51]

This demonstrates a broader problem of navigating fragmented care across services. Taylor et al. [36] show that after pancreaticoduodenectomy, many participants in their study were unsure whether to approach their GP, surgeon, oncologist, or specialist nurse. One participant recalled, “I don’t really know where I stand now, whether I see my GP or the oncologist or [surgeon].” Khan et al. [19] and Griffioen et al. [55] similarly demonstrate that the practical coordination of care often depended heavily on patients and carers, while Ploukou et al. [53] confirm from the provider perspective that patients need multidisciplinary, psychological, and symptom-focused support that is rarely straightforward to deliver or access. Compounding this is the lack of awareness of support that is out there, with participants in Khan et al. [19] stating patients “don’t know what exists or doesn’t exist”, and “don’t know if there’s a whole range of support services out there that (they are) just ignorant about.” An additional consequence of this fragmented care is the time toxicity of attending multiple providers on different days, in different locations, with one care partner stating, “There is so much time that is filled with all that the disease entails.” [55].

In several studies, participants described information, services, and supportive interventions that became apparent only after extensive self-directed searching, uncertainty, distress, or prolonged suffering. Taken together, these studies suggest that after the first urgent decisions are made, patients and families must continue to make sense of symptoms, appointments, treatment consequences, and recurrence risk, often within healthcare systems that assume levels of health literacy, stamina, social support, and organisational capacity that serious illness may have already eroded [19,29,36,44,51,53,55]. Additionally, these assumptions may be especially problematic for people already disadvantaged by social and structural determinants of health, who may have even less capacity to absorb this navigational burden.

### 3.3. Theme 3. “Things are not like they used to be…”: Embodied disruption, relational change, and reworking everyday life

The physical experience of pancreatic cancer was not reducible to isolated symptoms. Across the studies, participants described clusters of pain, fatigue, appetite loss, weight loss, altered bowel function, nausea, weakness, and digestive disruption that penetrated ordinary routines and restructured daily life. Mills et al. [47] show that even before diagnosis, changes in appetite, bowel habit, pain, and weight loss were central to how people gradually reinterpreted their bodies. After diagnosis and treatment, these issues often became even more prominent. Tang et al. [26] identify pain and fatigue as among the most pervasive and life-changing symptoms in advanced disease, while Liu et al. [37] suggest that postoperative patients often recognised only the most obvious symptoms, despite living with broader clusters that affected sleep, mood, appetite, and recovery. Symptom burden was therefore cumulative and relational. Its significance lay not simply in severity but in how symptoms interacted with one another and with the demands of everyday living and what these symptoms meant to people [19,26,35,37,40,47,53].

Food and eating emerged as one of the most disrupted aspects of everyday life. Studies focused on nutrition and exocrine insufficiency repeatedly show that eating shifted from a source of pleasure, routine, and sociability to a source of uncertainty, apprehension, and distress. This disruption occurred as participants found themselves needing to navigate digestive symptoms, weight loss, dietary restrictions, learning how to adjust PERT to different foods, and their bodies’ unpredictable responses. In Gooden and White [17], participants described persistent diarrhoea, pale and foul-smelling stools, burping, bloating, and the frustration of not knowing which foods triggered problems. One patient stated, “Diarrhoea, always diarrhoea!” In Cooper et al. [46], patients described “struggling with weight loss,” feeling pressured to eat, and experiencing food as both necessary and aversive. Landers et al. [52] and Dunleavy et al. [48] show that even when pancreatic enzyme replacement helped, it did not simply restore normality; instead, eating became tied to new routines, new expertise, and ongoing bodily monitoring. Milliron et al. [42] extend this picture by showing how nutritional care became a shared emotional burden, particularly for caregivers who felt responsible for calorie intake and distressed when weight did not increase. Across these studies, nutrition was not merely a clinical issue. It was intertwined with responsibility, identity, care, and the fear that bodily decline might signal treatment failure or disease progression [17,19,36,42,46,48,52].

Additionally, this remaking of eating altered family relationships and social participation. Relatives often encouraged, monitored, or pressured patients to eat, sometimes supportively and sometimes in ways that heightened distress. In Chauhan et al. [39], one participant said:

“I have stopped attending wedding functions because I need to be mindful of what I eat and drink there. I also avoid going because everyone asks the same question about what happened, which frustrates me.”

In Cooper et al. [46] and Milliron et al. [42], food became a focal point for family concern, guilt, and negotiation. Mills et al. [47] suggest that family members were already implicated in symptom interpretation before diagnosis, while Gooden and White [17] explicitly identified carer distress as part of the quality-of-life impact of pancreatic exocrine insufficiency. What these studies collectively suggest is that food occupies a particular place in pancreatic cancer because it condenses multiple losses at once: loss of appetite, loss of bodily reliability, loss of shared mealtime normality, and loss of confidence in how to care well. Nutritional problems are therefore not adequately understood as side effects alone; they are lived disruptions of domestic life, social participation, and caregiving relationships [17,19,39,42,46,47,52]. However, there is little attention paid to how people’s experiences of food and nutrition are shaped by their socioeconomic circumstances, food security, and access to practical and financial support. Across the studies, adapting to dietary changes, managing symptoms, and learning how to manage food digestion with PERT is largely presented as work undertaken by people and their families. Although several studies highlight inadequate information and support, few consider how wider socioeconomic factors influence people’s ability to manage these challenges. For example, it is broadly acknowledged that many people “cannot go to work or do household chores…” [53], and one participant in Chauhan et al. [39] describes their financial reality over the longer term:

“Experiencing some financial difficulties, especially after being at home for 2 months. I’m facing financial issues now. I’m not receiving any pay, and my medical benefits have run out.”

Some participants described receiving practical support with groceries, and family, friends, and community networks bringing them meals [41,44]. This raises an important question: if participants who described access to practical, financial, and/or social support during their illness still found it challenging to negotiate food and nutrition and manage related symptoms, how much more difficult might this be for people experiencing poverty, unstable housing, food insecurity, or social isolation? Therefore, how socioeconomic disadvantage specifically influences food security and nutritional coping in pancreatic cancer remains largely unexamined.

Broadly, participants described learning to live with compromised health: fatigue, weakness, postoperative pain, treatment toxicity, and bowel unpredictability restricted mobility, work, self-care, travel, hobbies, and sociality [17,20,35,39,41,48,53]. Tang et al. [26] show how pain affected activity and emotional well-being. Chauhan et al. [39] describe the burdens of travel, waiting, pain, and weakness during prolonged treatment. Khan et al. [19] and Wong et al. [14] discuss the effects of being “incapacitated from surgery”, confinement, and dependency on others: “you lose that sense of self.” [14]. A participant in Khan et al. [19] chimed with this, reflecting on the impact of fatigue, experiencing this for a long time after finishing treatment:

“I mean, I couldn’t even shower myself. I was out of breath. It was terrible. I had no one helping me… house cleaning and things like that. I couldn’t do that for months on end.”

Similarly, Ploukou et al. [53] reported that patients often cannot work or manage household tasks because diarrhoea, poor diet, pain, and pronounced fatigue undermine physical and social functioning. Financial toxicity was another consequence of this, with participants of working age discussing the financial hardship experienced, especially when they could no longer work [19,20,38,41,44] or had issues with their medical insurance [41,53] or receiving financial aid [19]:

“Previously, I used to receive pay along with medical benefits, but now that I’ve been at home for 2 months, my medical leave has also expired.”

[39]

Participants, particularly those who were of working age and employed (or the care partners of those who were), spoke of the financial strain caused by their pancreatic cancer, due to their inability to work [20,38,44], and the strain and worry this caused particularly in relation to their relationships with spouses and others [19,20,39,53]:

“My husband is the primary source of income in our family, but now he is unable to work…so the financial pressure is quite significant.”

[38]

Many expressed difficulties in accessing the support they needed, for example from social workers, in relation to financial aid and insurance cover [19,38,39,53].

“There’s not enough social workers; you’ve got to wait in line. Even when I was in the hospital and I asked for a social worker, there was no one available.”

[19]

However, support, particularly from family, friends, faith communities, and employers, mitigated this for others [38,39,41,44,54]. One participant in Stevens et al. [41] stated, “the work thing didn’t come into play” for them as they were retired, and Deobald et al. [29] recommend “the frequency of follow-up and travel were not major challenges for most patients as most were retired or could easily take time off work” but acknowledged the impact of “wait times at the clinic and parking costs”. This is ratified by Stevens et al. [41] too, as a retired participant spoke of the impact of transport costs in the context of limited pension income and family support. Beyond this, many studies included in this review did not delve into the financial impact of pancreatic cancer in any great depth and aside from mention of things such as “medical benefits” and “medical leave” in the context of India [39], “difficulties in accessing government-funded financial aid” in the context of Australia [19], “financial and insurance issues arising from the disease” in the context of Greece [53], “being on disability” [44] in the context of the USA, and “pension insurance” in the context of China [38], the impact of different healthcare and welfare models on financial toxicity is difficult to determine.

A gap noted is that although some studies discuss the impact of pancreatic cancer on household income, studies we have included in this review don’t explore the financial impact of pancreatic cancer in terms of care partners needing to take on most of the weight of the “chores around the house” [41] and responsibility for household income at a time when the person with pancreatic cancer also requires caregiving support, particularly during treatment and at the end of life, as a physician in Ploukou et al. [53] attests:

“Usually, the caregiver is needed from the beginning of the treatments to take care of financial, administrative, and social issues that arise. With the progression of the disease, in the last months of life, the patient needs a caregiver all day long.”

Participants found these role changes within the household and at work difficult [14,19,20,35,38,47,49,52,53], with some describing a lack of support from family [35,39] or facing criticism and conflict with family and friends over their decisions around finances [39], treatment [26,52], and diet [17], and contention around role changes at home and work [49]. Some studies discussed how pancreatic cancer strained relationships [17,20,26,35,39,49].

“You’re exhausted all the time and you can’t cope… so you’re lashing out at people.”

[35]

In contrast, others spoke of how their experiences navigating pancreatic cancer together strengthened their relationships with loved ones, particularly spouses [20,35,36,38,39]. One participant in Taylor et al. [36] spoke of how critical that support had been for them:

“It’s been tough for her as it has for me, but we’ve hacked through it together… I’ve got somebody to talk to and my wife will keep me in check, “have you done this, have you done that.” She’s fantastic. So that is the major part of it. I would feel for somebody who didn’t have that kind of backup.”

Ristau et al. [20]’s grounded theory on coping in people with pancreatic cancer concurs with this split, stating:

“Participants regularly reported that either couple relationships intensify and the partners try to overcome this crisis together, or they feel misunderstood by their closest relatives. The latter can especially be the case when patients have accepted their palliative state but relatives are not (yet) able to do so.”

Together, these studies indicate that pancreatic cancer did not simply affect the individuals diagnosed but often reconfigured their relationships and family lives. For some, relationships were strained by changes to household roles, caregiving responsibilities, financial pressures, and disagreements about treatment or the future. For others, navigating pancreatic cancer together strengthened their emotional bonds and increased their sense of mutual support. Rather than being static, relationships were continuously renegotiated as people adapted to the evolving challenges of living with pancreatic cancer.

Several studies suggest that patients gradually adapted to these limitations rather than simply measuring their lives against pre-illness standards. One participant reflected:

“And you see the last holiday we booked I ended up having to cancel it because I wasn’t well enough to go so… things are not like they used to be… It’s something you’ve got to get used to, I suppose, really.”

[35]

In these, people describe satisfaction with, or at least adaptation to, a “new normal” despite persistent symptoms [21,35,40,51], with a participant in Liu et al. [37] stating, “I’m used to it now”. Similarly, others suggest that recovery does not mean return to a former self so much as ongoing adjustment to altered capacity [20,21,35,44], although many were still “struggling to come to terms with” the impact of bodily and role changes [14,21,35,36].

Wancata et al. [21] show that many patients only fully understood treatment effects after experiencing them. Although most participants in Cooper et al.’s [46] study perceived their weight loss and reduced strength or stamina negatively, one participant who was previously overweight felt the weight loss was beneficial: “I felt better than I had done for years… and I’ve managed to keep it off”. The body that emerges in these accounts is therefore not only suffering but learning, recalibrating, and enduring under chronic strain [21,26,35,37,39,40,46,53].

Across the studies, fatigue, bowel problems, dietary restrictions, appetite loss, and pain mattered not only because they were physically burdensome, but because of how they affected autonomy, social identities, intimacy, relationships, and social participation. Symptoms such as digestive problems, weakness, appetite loss, and pain acquired meaning through the ways in which they disrupted everyday life, challenged personal dignity, and altered roles and participation in valued activities, such as going out for a meal with loved ones. These findings suggest that symptom burden and psychosocial impact were closely entwined. Symptoms were experienced not simply as physical effects of their cancer or cancer-related treatment(s) but were contextualised by their consequences for people’s social identities, relationships, independence, and participation in everyday life [17,19,26,35,37,46].

### 3.4. Theme 4. “Living on borrowed time”: Coping with uncertainty and mortality

If diagnosis was experienced as a threat to life itself, ongoing treatment and survivorship did not resolve that threat for participants. Instead, many speak of learning to live with ongoing uncertainty around recurrence, progression, and survival. Several studies make clear that participants did not move neatly from crisis to recovery. Instead, they lived in repeated cycles of vigilance, hope, fear, and recalibration in relation to how much time they might have to live and what the future might hold. Ristau et al. [20] describe this tension as holding “the question about the remaining time of life” alongside a “simultaneous awareness of one’s finiteness”. They describe participants as moving between “hopes and wishes on the one hand and fears and concerns on the other” while follow-up investigations repeatedly retriggered uncertainty:

“The whole life of people with pancreatic cancer is repeatedly endangered by follow-up examinations, which they perceive as an acute threat, as each carries the possibility of discovering that the cancer has progressed or recurred.”

[20]

Winebrenner et al. [43] provide the clearest account of this in describing “the recurring cycle of scanxiety,” where surveillance scans triggered anticipatory worry long before appointments occurred. One participant explained, “The date gets locked in my brain. It’s always there looming over me.” Related patterns appear in Dengsø et al. [51], where CA-19-9 results produced “ambiguous reassurance,” and in Deobald et al. [29], where follow-up provided comfort precisely because it held open the possibility of detecting recurrence. Taylor et al. [35] identified the burden of monitoring and new symptoms after surgery, while Bleicher et al. [40] noted heightened anxiety around surveillance imaging and tumour markers. Collectively, these findings show that post-treatment life is structured by repeated returns to uncertainty rather than steady movement away from it [29,35,40,43,51].

Within that uncertainty, participants developed varied and sometimes contradictory ways of coping. Bleicher et al. [40] explicitly identified depression, acceptance, denial, positive thinking, and a fighting attitude as coexisting orientations. Ristau et al. [20] conceptualised coping as movement from surviving the acute threat towards “living on,” in which the task becomes not only to stay alive but to re-establish some meaningful way of inhabiting life under the shadow of mortality. Frenkel et al. [54] similarly showed that exceptional survivors often did not think of themselves as cured; instead, survival was understood literally, as continuing to live despite ongoing symptoms and vulnerability. Hope in these studies was rarely naïve optimism. Rather, it was repeatedly reframed. Sometimes it meant cure or remission, but just as often it meant time, routine, family presence, or the possibility of a good day. In Bleicher et al. [40], patients valued “just my regular routine,” while Frenkel et al. [54] emphasised gratitude for ordinary existence. Even in Winebrenner et al. [43], where scanxiety was intense, hope persisted through efforts to “reset the clock” after reassuring results.

“When ‘he says in three months time, I’m going to see you again . . . it’s giving you hope that everything is going to be okay.”

[29]

These accounts suggest that hope in pancreatic cancer is dynamic and often modest, but no less significant for that [20,38,40,42,43,44,49,50,54]. Participants similarly spoke of their faith and spirituality as important coping resources. For some, religious faith helped to sustain hope, find meaning in their experiences with the disease, and live with the uncertainty associated with a pancreatic cancer prognosis. Faith provided a framework within which people could make sense of their illness in relation to concepts such as divine will, divine control, or destiny [34,39,41,44,54]. One participant in Stevens et al. [41] stated: “…my wife and I, we are Christians and we know it’s up to him, the Lord.” Several participants described placing their trust in God, expressing gratitude for continued life, or viewing each day as a gift despite the uncertainties associated with pancreatic cancer [44,54].

Beyond close relationships with family and friends, participants described the supportive role of faith communities, community organisations, support groups, and healthcare professionals. These avenues were described as providing emotional reassurance, practical assistance, information, opportunities to connect with others facing similar experiences [19,36,41,44], plain-language information, and signposting to support services [19].

At the same time, coping was clearly relational. Family members, spouses, friends, faith communities, and clinicians could help patients emotionally, but these relationships also carried burden, guilt, and protective silence. For those who had supportive relationships, care partners were indispensable for emotional support and companionship [14,19,35,38,39], advocacy and assistance navigating healthcare systems and treatment decisions [35,36,41,49,50,54], transport to appointments and treatments [41], information management and interpretation of medical information [35,36,41,44,48,54], monitoring symptoms and changes in health status [45,53], medication management (particularly PERT) [48,52,53], meal preparation and nutritional support [17,42,45,54], and practical support with housework [41,53]. Care partners who had healthcare backgrounds sometimes played an additional role in helping patients understand complex information, access services, interpret symptoms, and navigate treatment decisions [44,54].

“My son works in the health field, at home he always told me if I had any doubts about something, it was much easier to understand. When something different happened, he would tell me to let him know.”

[44]

In Zhang et al. [38], however, patients and care partners sometimes withheld information, concerns, and emotions from one another to protect each other from further distress [35,51]. Participants experienced guilt and worries about leaving loved ones behind [34,35,36,41], particularly their children [26,35], and sometimes expressed fears of being forgotten [26].

Wong et al. [14] and Taylor et al. [35] show that partners were themselves profoundly distressed, sometimes more visibly so because they could not intervene directly in the care of the person with the disease. In advanced illness, Chapple et al. [45] highlight the importance of home, comfort, dignity, and the one-to-one care that spouses often provided. These studies therefore resist any simple distinction between patient experience and caregiver experience. Where relevant, pancreatic cancer may reorganise the family, where it exists, as a unit of practical, emotional, and existential support [14,17,19,35,36,38,41,42,45].

The psychological burden of not wanting to burden others recurred across the dataset [35,36,38,44,45,51,52]. It influenced participants’ decisions on their preferred place of death, with participants in Chapple et al. [45] deciding to rent out accommodation to die in, or opt for hospital or hospice instead to protect their loved ones from associating their homes with their deaths:

“I don’t care if I’m in a little corner of the hospital as long as I’m not here [at home]”. I do not want to be here (. . .) I do not want the kids walking in and saying, ”Oh, that’s where my Mum died,” or like my husband getting in bed and thinking, ’Oh, my wife died in here with me’.”

It also meant participants sometimes “downplay(ed) their pain and symptoms” [52], avoided asking for help, and withheld fears to protect spouses and relatives already under strain:

“(A)t home I avoid talking about my cancer. Because it makes (Name) sad if I say anything. He gets sad, so I’ve simply stopped talking about it. I never talk about my cancer at home. I never talk about my anxiety and fears. I don’t talk about it. I don’t talk about it.”

[51]

“[Husband] always comes with me to the appointments and I said to him yesterday ‘where are you going to sit’, because I thought I’m not, I can’t speak openly about things because I don’t want to upset [him].”

[35]

Taylor et al. [36] describe patients feeling both “a burden and being burdened,” especially when primary care support felt uncertain. Zhang et al. [38] capture this directly in the statement, “I certainly hope she can help me, but it is already very difficult for her, and I don’t want to burden her even more.” Similar tensions appear in Milliron et al. [42], Liu et al. [37], and Gooden and White [17] around food, nutrition, and caregiver guilt, and in Ploukou et al. [53] through clinicians’ recognition that caregivers take on increasing administrative, practical, and emotional work as disease progresses. This matters analytically because it shows that psychosocial suffering is often displaced into efforts to remain stoic, cooperative, or undemanding. Need, in other words, is frequently hidden inside attempts not to add to others’ need [17,36,38,42,44,53].

Across these studies, living with pancreatic cancer therefore appears less as a stable state of recovery and more as an ongoing practice of negotiating mortality, symptoms, relationships, and incomplete reassurance, with one salient example from Taylor et al. [35] stating:

“You feel you’ve been re-defined and you don’t know what your future is or what your future is going to be and the diagnosis and the prognosis measures your life out for you.”

The psychological work involved in navigating that uncertainty is cyclical rather than linear, and relational rather than purely individual. Patients often live from scan to scan, marker to marker, and symptom to symptom, repeatedly recalibrating hope downward from potential cure toward continued life, manageable distress, family presence, or ordinary routine:

“I want to grab what I can now. Hopefully these pains will get a bit better, and I can do a bit more. I really felt quite optimistic last week; in fact, I suppose I’m angry because I was quite high on Monday and yesterday I was quite low. Yesterday I suddenly thought I’m not going to get the drug. I’m going to die soon. Monday, I was going to live…”

[22]

Yet, that recalibration should not be mistaken for resignation. It is better understood as a disciplined form of adaptation through which people continue to make life possible under conditions that remain profoundly uncertain [14,20,29,34,35,38,40,41,43,44,49,51,54].

## 4. Discussion

This review suggests that for the people recruited to the included studies, the psychosocial experience of pancreatic cancer is best understood less as a linear process and more as an ongoing and fluctuating negotiation of mortality, uncertainty, symptoms, relationships, and everyday life. In many of the included studies, participants described periods where their awareness of death—specifically their proximity to their own death—increased in response to concerns about cancer recurrence or disease progression and decreased in response to reassuring test results or periods of relative stability. Factors provoking these concerns included the development of new symptoms, follow-up scans, and blood tests.

One framework these findings could be interpreted through is Terror Management Theory (TMT) [58]. From this perspective, mortality awareness can affect how people respond to a serious illness like pancreatic cancer and attempt to preserve meaning, connection, hope, and a sense of everyday continuity. Several aspects of the synthesis align with this framework; as outlined in the previous paragraph, participants described periods where awareness of their mortality became more pronounced (e.g., approaching scan appointments) and less pronounced (e.g., receiving reassuring test results). Furthermore, they described efforts to cope with and manage the uncertainty and psychological distress associated with living with pancreatic cancer. These strategies included denial and avoidance [20,22,36,41,43,51], relying on social relationships for emotional support [19,20,22,36,38,39,44,51], engaging with faith or spirituality [34,39,41,44,49,54], and making meaning out of their experiences [21,35,42,44,51,52,54].

In that sense, the findings align with a broader body of scholarship across psycho-oncology, palliative care, supportive care, nursing, and oncology, recognising that cancer care is not only a technical and biomedical enterprise, but a profoundly human experience shaped by fear, meaning, identity, relationships, and the anticipation of loss [59]. They further resonate with the concept of “double awareness”, in which people attempt to remain engaged in life while simultaneously preparing for possible death [60]. Ristau et al.’s [20] grounded theory reflects this double awareness, stating that people hold “the question about the remaining time of life with simultaneous awareness of one’s finiteness”. Similarly, a participant in Winebrenner et al. [43] expressed the need to “balance being a realist with kicking myself in the butt to keep going. I have to remind myself, you’re not dead yet!” Seen in this way, the present synthesis does not merely describe distress; it shows how people repeatedly attempted to keep living meaningful lives while adapting to symptom experiences, changing roles and identities, uncertainty around mortality, and the possibility of approaching death.

Pancreatic cancer may intensify these processes due to its poor prognosis, typically late presentation, and advanced stage at diagnosis, which often leaves limited time for people to process and begin to acknowledge the emotional and practical consequences of their diagnosis before navigating treatment decisions, symptom escalation, or disease progression. That context matters for interpretation of the qualitative findings. It helps to explain why diagnosis was experienced in the included studies not simply as bad news, but as existential rupture; why participants often described treatment as urgent rather than elective; and why uncertainty persisted even in survivorship. Similar themes have been reported in incurable oesophagogastric cancers, where people strived for normality within unpredictable and threatening situations [61]. However, pancreatic cancer may be distinctive in the extent to which vague pre-diagnostic symptoms, compressed timelines, and persistent gastrointestinal symptom burden often combine to limit the time and space available to individuals to process and respond to their diagnosis.

Even among individuals who are candidates for treatment with curative intent, uncertainty often persists. Many people with cancer in general report experiencing fear of cancer recurrence, with 59% experiencing a moderate amount of this fear, and 19–22% experiencing levels high enough to warrant specialised intervention [62]. In pancreatic cancer, one study reported roughly 37% of patients experienced clinically significant fear of cancer recurrence following potentially curative pancreatectomy for adenocarcinoma, regardless of their actual risk of recurrence [63]. These findings suggest that some psychosocial challenges (such as fears of recurrence and progression, uncertainty, and awareness of mortality) which are shared by different cancer populations might be especially salient in pancreatic cancer given its low survivability. The findings indicate what may be required is not just an understanding of psychosocial needs specific to a diagnosis of pancreatic cancer, but models of care that can recognise the realities of compressed treatment and supportive care timelines, complex and severe symptoms, ongoing uncertainty, concerns around recurrence and progression, and shifting support needs across the illness trajectory. Thus, these findings have implications not just around what support is accessible to people, but how support is organised and delivered across the illness trajectory.

Although this review was not carried out from a psychotherapeutic perspective, several findings closely align with concerns addressed by psycho-oncology interventions. One example is the psychotherapeutic intervention ‘Managing Cancer and Living Meaningfully’ (CALM) and related existential models in the field of psycho-oncology [64]. CALM was developed specifically for people with advanced cancer and attends to four domains that map strikingly well onto the themes in this review: symptom management and communication with healthcare professionals; changes in self and close relationships; spirituality or meaning; and thinking about the future, hope, and mortality [59,64]. The current synthesis suggests that all four are highly salient in pancreatic cancer. Participants were not only burdened by symptoms, but by the meanings attached to those symptoms and were concerned with protecting others from distress. Furthermore, participants were not only fearful of death but engaged in active reworking of hope towards more immediate and achievable goals. This suggests that CALM might offer a useful framework through which to understand and respond to participants’ concerns—not because it offers a generic psychotherapy solution, but because it directly addresses many of the concerns described by participants, including uncertainty about the future; fears of recurrence and progression; changes in roles, identities, and relationships; coping with the impact of symptoms; and searching for meaning and hope.

Likewise, meaning-centred psychotherapy may be relevant where existential threat, altered identity, and questions of purpose are prominent [65]. The implication here is not that every person with pancreatic cancer requires formal psychotherapy or specialist psychological intervention. To be more exact, the findings of this review indicate that existential distress and concerns, fears of recurrence and progression, and changes to identity and roles (and associated relationship challenges) are commonly experienced aspects of living with pancreatic cancer that could benefit from being highlighted and discussed routinely within multidisciplinary care. The challenge for services is not, therefore, to provide specialist psychological interventions to everyone, but instead to identify the changing needs of people and facilitate timely access to the appropriate support when it’s desired or needed.

The review highlights how uncertainty is further produced by the organisation of care. Participants described significant informational labour around treatment decisions, diet, PERT, follow-up, and surveillance. This is important because it suggests that uncertainty is not simply an internal psychological state; it is shaped by gaps in communication, fragmented follow-up, and the burden placed on patients and families to coordinate care. Existing pancreatic cancer literature supports this interpretation. UK survey data show substantial unmet supportive care needs, especially around information and support [66]. Qualitative work on post-resection surveillance demonstrates that follow-up can be both reassuring and anxiety-provoking [29], while recent work on “scanxiety” in pancreatic cancer survivors demonstrates how surveillance can create a recurring cycle of anticipatory distress [43]. Likewise, practical guidance on pancreatic exocrine insufficiency emphasises that gastrointestinal symptoms, malnutrition, and quality-of-life consequences require proactive recognition and management rather than ad hoc advice [57]. Collectively, these sources suggest that psychosocial care in pancreatic cancer should include clearer, repeated, and better-timed communication about symptoms, nutrition, pancreatic enzyme replacement therapies, surveillance, and routes back into care.

Given the significant gastrointestinal symptom burden associated with pancreatic cancer and its treatments, and the psychosocial impact of this, these findings highlight the importance of timely access to specialist dietetic and nutritional support. In particular, participants’ experiences suggest the value of input from professionals with expertise in pancreatic cancer and pancreatic exocrine insufficiency to support the recognition and management of nutritional difficulties and symptoms of PEI through appropriate use of PERT. Similarly, the findings support proactive pain assessment and timely access to pain management and palliative care specialists where required, particularly when pain is severe, persistent, or difficult to alleviate. Such multidisciplinary approaches may help to address not just the physical burden of symptoms, but their effects on quality of life, psychological wellbeing, everyday functioning, and relationships with loved ones too.

Early qualitative work by Fitzsimmons et al. [22] highlighted that healthcare professionals and people diagnosed with pancreatic cancer sometimes interpreted quality of life differently, with professionals placing more emphasis on symptom burden and physical functioning, whereas patients emphasised how symptoms related to their identities, relationships, hopes, and sense of meaning. However, these findings should be interpreted within the context of the substantial developments the past 27 years have brought, which have built on that longer-standing recognition, also evidenced in research on other upper gastrointestinal cancers at the time [67], that quality of life is a meaningful outcome beyond survival time. These include increased importance placed on person-centred care, supportive care screening, multidisciplinary cancer services, psycho-oncology, specialist nursing roles and practice, care navigation, and patient-reported outcome measurements. Therefore, our findings, particularly those presented in Theme 3, do not necessarily imply a divide between patients and healthcare professionals. Instead, they suggest that symptoms continue to draw much of their significance from the effects they exert on everyday life, relationships, identity, and perceptions of the future.

Another important finding is that distress in pancreatic cancer is often relational. Patients in the studies included in this review were frequently attempting to manage not only their own emotions but those of partners and families too, sometimes by withholding fears to protect others. This relational pattern is highly consistent with emerging pancreatic cancer caregiver literature; a recent scoping review found that caregivers commonly experience stress related to caregiving tasks, disruption to daily life and relationships, unmet needs, poor QoL, and a persistent sense of inadequate support [68]. Interdependence between patient and care partner psychosocial wellbeing is well supported in psycho-oncology, especially in relation to distress, QoL, and related dyadic outcomes [69,70]. This review’s findings reinforce the extent to which care partners’ wellbeing can be intertwined with patients’ own emotional experiences and coping processes. Clinically, this highlights the importance of considering the psychosocial support needs of not just individuals but the wider support network around them, since patients’ emotional wellbeing and distress can be influenced by the wellbeing and emotional responses of their partners, family members, and other significant people in their lives.

These findings support consideration of family-based or dyadic approaches for some people, especially at key transition points such as diagnosis, treatment changes, discharge home, and surveillance appointments. However, it is important to recognise that patients’ relationships and sources of support can be varied, and the involvement of partners or relatives may not be appropriate or desired in all circumstances. Some individuals might have limited social support or experience strained or unsafe relationships. Therefore, psychosocial support should be guided by people’s preferences, relationship dynamics, and considerations of safety and wellbeing. Care and support should take into account the wider social context of people’s lives, recognising that meaningful supportive relationships might look different from individual to individual, and may extend beyond, or exist independently of, family relationships. This broader understanding of support is reflected in our use of the term “care partner”, rather than “family caregiver”, acknowledging that significant sources of support might include friends and neighbours, as well as other significant relationships and relatives. A caveat to this is that, while the psychosocial interdependence between patients and care partners is well established, most evidence describes spouses, partners, and family care partners. In comparison, the psychosocial interdependence between patients and other significant relationships is less explored. Comparable evidence from incurable oesophagogastric cancer demonstrates that relatives try to manage the disease “together but still alone”, reinforcing the importance of shared but differentiated support across gastrointestinal cancers with poor prognosis [71].

In practical terms, these findings support a model of care in which psychosocial support is proactive, repeated, and integrated into routine pancreatic cancer care rather than reserved for crisis referral. Distress screening guidance in oncology has long recommended systematic identification and management of psychological or generalised distress, but the current synthesis suggests that a one-off assessment is unlikely to be adequate in pancreatic cancer because psychosocial needs are dynamic and change across the illness trajectory [72]. This is consistent with recent work from Pancreatic Cancer UK [73] proposing an Optimal Care Pathway which recommends proactive access to psychosocial support services, nutritional management, rapid access symptom support, and better post-resection follow-up, as well as European advocacy work [74] which positions emotional care as a central element of comprehensive pancreatic cancer care. Alongside face-to-face psycho-oncological input, there may be scope to explore innovative psychoeducational and digitally delivered approaches; a recent scoping review of evidence on serious games in cancer suggests that interactive formats can support education, engagement, and behaviour change, although pancreatic cancer-specific intervention evidence remains limited [75].

The stark uptick in qualitative research in pancreatic cancer in recent years (26/30 included studies were published in the past decade, as per Figure 2) could be attributed to concurrent developments in the fields of pancreatic cancer and qualitative research. There appears to be an increase in recognition of the importance of improving quality of life, supportive care, and psychosocial outcomes for a cancer with such poor survival and high symptom burden [13,16,76,77,78], perhaps due to international advocacy efforts and investment in pancreatic cancer research. In tandem with this is the growing acceptance and promotion of qualitative research methodologies within oncology and health research, leading to an increased study of patients’ lived experiences, alongside traditional biomedical outcome measures captured in quantitative research [79,80]. However, despite these developments, important gaps remain around whose experiences are represented and how these are captured and understood.

Therefore, the review identifies clear research priorities. The literature remains scarce for people diagnosed at an advanced stage with no treatment options with curative intent, for those approaching end of life, and for families living through short and rapidly changing trajectories. Longitudinal qualitative research would be particularly valuable to examine how mortality awareness, coping, relationships, and informational needs change over time. Moreover, there is a strong case for developing and testing pancreatic cancer-specific psychosocial interventions that are sensitive to symptom burden; anxieties around recurrence, progression, and death; gastrointestinal and nutrition-related distress; and family impact. In that regard, the authors’ protocol for a grounded theory study in Northern Ireland is well positioned to extend this review by generating a more holistic account of the pancreatic cancer journey across diagnosis, treatment, survivorship, and end of life [81]. Overall, the present review makes a persuasive case that pancreatic cancer should not be treated as merely another cancer site to which generic psychosocial models can be applied. Its psychosocial reality is shaped by a distinctive combination of symptom ambiguity, diagnostic shock, embodied disruption, relational strain, and persistent confrontation with mortality. These implications extend beyond our psychosocial understanding of the disease to the organisation of care itself, particularly in a global climate of increasingly resource-constrained healthcare systems, where the need is not simply for more support, but for timely, coordinated, equitable, and efficient individualised care.

Looking forward, we anticipate that qualitative research will be critical to understanding how people experience pancreatic cancer in the context of emerging developments such as KRAS-targeted therapies and other precision-oncology approaches [82], immunotherapies, and efforts in screening and earlier diagnosis [83], particularly since these developments could substantially affect survival and illness trajectories for some people. Understanding how new treatments influence the psychosocial aspects of pancreatic cancer will be crucial to ensuring that progress in this field is measured holistically—not simply quantifying the number of months lived but capturing how those months are experienced too.

A further implication of this review concerns whose experiences are represented in the available evidence base. The studies included largely present the experiences of people with pancreatic cancer who were able to access healthcare, engage with support services, and participate in research, raising the critical question of whose voices may be absent from the literature. There are likely groups who are underrepresented in these studies, if at all, such as people from low-income countries; people from lower socioeconomic groups; people living with severe mental illness; and those who do not receive as much support or information and who do not feel able to participate in research due to symptom burden and psychological distress, even if they are aware that research exists. We already know that socioeconomic disadvantage negatively influences outcomes in pancreatic cancer, resulting in more advanced disease at diagnosis, reduced access to surgery, and poorer outcomes even post-treatment; this suggests that psychosocial experiences could also differ across socioeconomic groups and healthcare contexts [84]. It is reasonable to consider that if the psychosocial aspects of pancreatic cancer are this challenging for people who do have access to care, information, treatment, and support, then these challenges may be even greater for those who are underserved and disadvantaged. Finally, as novel therapies emerge onto the pancreatic cancer landscape, future research should ideally seek to capture who is able to access these advancements and assess whether their benefits are experienced equitably across different populations.

## 5. Strengths and Limitations of the Review

This review has several strengths. To our knowledge, it provides the most up-to-date qualitative synthesis of the psychosocial experiences of people living with pancreatic cancer across the illness trajectory, from symptom onset and diagnosis through treatment, survivorship, and advanced disease. In doing so, it extends previous qualitative syntheses that focused mainly on psychological experiences or living with pancreatic cancer by adopting a broader psychosocial perspective and including a substantially expanded and more recent evidence base. The review was prospectively registered in PROSPERO and reported using established systematic review guidance. A comprehensive search was undertaken across seven bibliographic databases, supplemented by reference list screening and forward citation tracking, which strengthens confidence that relevant literature was identified. Study selection, full-text screening, and methodological appraisal were undertaken independently by two reviewers, and thematic synthesis was conducted systematically using Thomas and Harden’s [33] approach. Together, these features support the transparency and rigour of the review process.

This review has its limitations. First, the included literature was heterogeneous in design, setting, and focus, and some studies contributed more directly to the review question than others. Second, although the synthesis captures experiences across the pancreatic cancer journey, people with very advanced disease, those receiving end-of-life care, and those with rapidly progressive illness appear to be under-represented, likely reflecting the practical and ethical challenges of conducting research with these groups because they are too unwell, or are considered to be too unwell, to approach. Third, although the review included international studies, the evidence base was weighted towards high-income settings, which may limit transferability to lower-resource healthcare systems or different cultural contexts. Furthermore, the included studies mostly represent the experiences of people who were able to access healthcare services and participate in research, potentially under-representing people facing barriers related to socioeconomic disadvantage, limited social support, disability, severe mental illness, or other social and structural determinants of health (discussed further above as a broader evidence gap). Although some studies reported relationship strain and included accounts suggestive of strained or unsupportive spousal and family relationships, few appeared to explore the quality, stability, or safety of participants’ relationships in depth. As a result, the synthesis may disproportionately reflect the experiences of people with access to supportive relationships, and the psychosocial experiences and support needs of those living with strained, limited, or potentially unsafe relationships remain underexplored. Finally, few studies reported participants’ comorbidities, socioeconomic circumstances, or access to healthcare resources, limiting our ability to understand how psychosocial experiences might vary across different social and healthcare contexts. These limitations should be considered when interpreting the findings.

## 6. Conclusions

This qualitative systematic review suggests that the psychosocial experience of pancreatic cancer is characterised by an ongoing and fluctuating confrontation with mortality, shaped by embodied disruption, relational dynamics, and persistent uncertainty. Rather than moving through a linear process of adjustment, people living with pancreatic cancer appear to remain in flux as they navigate diagnosis, treatment, symptom burden, surveillance, and fears of progression, recurrence, and death. The review highlights that psychosocial distress in pancreatic cancer is not confined to isolated moments of crisis but rather is woven through the illness trajectory and closely linked to communication, symptom management, family relationships, and perceptions of the future. Recognising this complexity is important for developing pancreatic cancer care pathways that integrate timely psychosocial support, clear and responsive information, and attention to the needs of both patients and those close to them. Further research is needed to strengthen understanding of under-represented groups identified throughout this review, and to inform the development of tailored psychosocial interventions in this population.

## Figures and Tables

**Table 1:** Study inclusion/exclusion criteria.

	Inclusion Criteria	Exclusion Criteria
Population	People with any pancreatic cancer diagnosis. People with perspectives and insights (care partners/family and professionals) on the psychosocial aspects of experiences of living and dying with pancreatic cancer where these focus on the person who has/had pancreatic cancer.	Animal/non-human participants People with multiple cancer diagnoses of which pancreatic cancer is one and it is not the focus of the study. People with pre-malignant diagnoses such as intraductal papillary mucinous neoplasms. Studies which collect data from people with insight into pancreatic cancer journeys (care partners or professionals) where they focus on their own experiences, rather than providing their perspectives of and insight into the psychosocial experiences of people with pancreatic cancer. Although caregiver wellbeing is known to influence patient wellbeing and coping, caregiver-focused studies were outside the scope of this review because they address a distinct research question and have been the subject of dedicated evidence syntheses.
Exposure	Literature exploring the psychosocial aspects of the experiences of diagnosis, treatment, palliative care, or survivorship with people who have a confirmed diagnosis of any pancreatic cancer at the time of data collection.	Literature which focuses on experiences of pancreatic cancer, such as the physical symptoms, without providing data on the psychosocial aspects of these (i.e., distress, anxiety, coping). Studies which focus on advanced cancers in general, where participants with pancreatic cancer form a small part of the overall population (less than 33%) and data and analysis of their data cannot be extricated from the rest of the sample. Literature where the exposure to pancreatic cancer is ‘incidental’ or considered a comorbidity and not the focus of the study, such as where a concurrent diagnosis of something else is the focus and it is not considered related to the pancreatic cancer diagnosis; for instance, where depression, anxiety, or relationship changes are experienced and related to the pancreatic cancer journey, this would be considered relevant to the research question. Intervention studies.
Outcome	Psychosocial aspects of experiences. In this context, “psychosocial” refers to the interaction between psychological, social, relational, and contextual factors which influence people’s ability to navigate and cope with the challenges of their cancer across the disease trajectory (diagnosis, treatment, survivorship, palliative, and end-of-life care). Therefore, psychosocial aspects incorporate a wide range of factors, including: Emotional and mental well-being (anxiety, burden, depression, distress, emotional processing, grief, hope, mood, post-traumatic stress, stress, and uncertainty). Benefit finding, coping and adjustment mechanisms (relating to treatment, symptoms and side effects, prognosis, and role, body, and lifestyle changes). Quality of life and wellbeing (including participants’ perceptions of the impact of pancreatic cancer on their daily lives and overall wellbeing). Social support and relationships (including experiences of discrimination and social stigma, and communication and interactions with health care professionals, family members, friends, colleagues, and their wider community). Financial toxicity and burden and its associated effects. Spirituality and faith (finding meaning, exploring existential questions, and how people navigate this when confronted with their mortality and a cancer diagnosis).	Physical aspects of the experiences of people diagnosed with pancreatic cancer where data is not provided for the psychosocial aspects of these. For instance, where a study looks at the experiences of someone following surgical treatment or neoadjuvant chemotherapy for pancreatic cancer, if it looks only at the physical aspects of these experiences (i.e., symptoms of pancreatic exocrine insufficiency and pain), then it will be excluded. However, if it also looks at the psychosocial aspects of these physical symptoms (i.e., distress, ability to cope, anxiety, depression, anger), then it will be included.
Study Characteristics	Studies with qualitative designs or mixed methods studies with extractable qualitative data Studies investigating or exploring the psychosocial aspects of living and dying with pancreatic cancer. Published, empirical articles and successfully defended dissertations (to ensure literature has undergone a peer review process).	Commentaries, letters, editorials, position statements, books and book chapters, and guidelines. Literature reviews and meta-analyses (included studies of relevant reviews will be screened). Conference proceedings and abstracts, but we will look for a published write-up of findings. Intervention studies.

## Data Availability

Supporting data may be made available by request to the corresponding author (L.C.).

## Supplementary Materials

The following supporting information can be downloaded at https://www.mdpi.com/article/10.3390/cancers18193177/s1: Table S1: PRISMA 2020 Main Checklist. Table S2: Search history and results; Table S3: CINAHL Complete (EBSCOhost) search strategy; Table S4: Embase (Ovid) search strategy; Table S5: MEDLINE (Ovid) search strategy; Table S6: APA PsycINFO (Ovid) search strategy; Table S7; Scopus search strategy; Table S8: Web of Science Core Collection search strategy; Table S9: WHO Global Index Medicus search strategy; Table S10: Study characteristics. Table S11: Reporting of socioeconomic demographics across studies.

## Appendix A

Appendix A—Table A1, Table A2, Table A3, Table A4, Table A5, Table A6 and Table A7: search terms for each database.

**Table A1:** CINAHL search terms.

PEO Element	CINAHL Subject Headings (MH)	Synonyms/Free Text Keywords	With Truncation/Wild Card Use
*Population (P):* *People diagnosed with pancreatic cancer*	((MH “Pancreatic Neoplasms+”) OR ((MH “Pancreas+”) AND (MH “Neoplasms+”))		“Pancrea* N4 cancer*” OR “Pancrea* N4 tumo#r*” OR “Pancrea* N4 carcinoma*” OR “Pancrea* N4 adenoma*” OR “Pancrea* N4 adenocarcinoma*” OR “Pancrea* N4 neoplasm*”
	(MH “Patients”) OR (MH “Cancer Patients”) OR (MH “Hospice Patients”)	People	Patient* OR (people W2 with) OR survivor* OR person*
*Exposure (E):* *experiences of living (and dying with) pancreatic cancer*	(MH “Anger”) OR (MH “Anxiety+”) OR (MH “Attitude”) OR (MH “Attitude to Death+”) OR (MH “Attitude to Health”) OR (MH “Attitude to Illness”) OR (MH “Bereavement+”) OR (MH “Coping”) OR (MH “Death”) OR (MH “Depression”) OR (MH “Emotions+”) OR (MH “Fear”) OR (MH “Grief+”) OR (MH “Health+”) OR (MH “Life Experiences”) OR (MH “Life Change Events+”) OR (MH “Psychological Distress”) OR (MH “Psychological Well-Being”) OR (MH “Psycho-Oncology”) OR (MH “Psychosocial Intervention”) OR (MH “Quality-Adjusted Life Years”) OR (MH “Quality of Life”) OR (MH “Self Concept+”) OR (MH “Spiritual Distress (NANDA)”) OR (MH “Spiritual Distress (Saba CCC)”) OR (MH “Support, Psychosocial”) OR (MH “Symptom Burden”) OR (MH “Symptom Distress”) OR (MH “Terminally Ill Patients+”) OR (MH “Wellness”)	Anger OR “Benefit finding” OR Bereavement OR Body image OR Burden OR Coping OR Cope OR Death OR Dying OR Grief OR Identity OR “Intrusive thoughts” OR “Positive affect” OR “Post-traumatic growth” OR “Post-traumatic stress” OR Psychosocial OR “Quality-of-life” OR Self-appraisal OR Self-concept OR Self-discontent OR Self-esteem OR Self-identity OR “Social support” OR Somatic OR Somatisation OR Wellbeing OR Well-being	OR Accept* OR Adjust* OR Anxiet* OR Attitude* OR Belief* OR (cognitive N3 function*) OR Depress* OR Discriminat* OR Distress* OR Emotion* OR (Emotion* N3 disturb*) OR Existential* OR Experience* OR Fear* OR Helpless* OR Hope* OR Hopeless* OR Intima* OR Journey* OR Lonel* OR Marriage* OR (Outlook N3 life) OR Perception* OR Perspective* OR Preoccupation* OR (Psycholog* N3 distress) OR (Psycholog* N3 need*) OR Psycho-oncolog* OR (Psychosocial* N3 need*) OR (Quality N3 life) OR Regret* OR Relationship* OR (Satisfaction N3 care) OR (Satisfaction N3 life) OR Sexual* OR Social* OR (Social N3 activit*) OR (Social N3 function*) OR (Social N3 support*) OR (Social N3 well-being) OR Spirit* OR Stigma* OR Survivor* OR (unmet N4 need*) OR Worr*

**Table A2:** Embase search terms.

PEO Element	Emtree	Synonyms/Free Text Keywords	With Truncation/Wild Card Use
*Population (P):* *People diagnosed with pancreatic cancer*	exp pancreatic neoplasm/ OR (exp pancreas/AND exp neoplasm/)		(pancrea* adj4 cancer*).m_titl. OR (pancrea* adj4 tumor*).m_titl. OR (pancrea* adj4 carcinoma*).m_titl. OR (pancrea* adj4 adenoma*).m_titl. OR (pancrea* adj4 adenocarcinoma*).m_titl. OR (pancrea* adj4neoplasm*).m _titl.
	exp patients/ OR exp persons/ OR exp cancer patient/ OR exp hospice patient/	People	patient* OR (people ADJ2 with) OR survivor* OR person*
*Exposure (E):* *experiences of living (and dying with) pancreatic cancer*	exp anger/ OR exp anxiety/ OR exp attitude/ OR exp attitude to death/ OR exp attitude to health/ OR exp attitude to illness/ OR exp bereavement/ OR exp psychological adaptation/ OR exp death/ OR exp depression/ OR exp emotions/ OR exp fear/ OR exp grief/ OR exp health/ OR exp life change event/ OR exp psychological distress/ OR exp mental health/ OR (exp neoplasms/AND exp psychology/) OR exp psychosocial care/ OR exp quality-adjusted life year/ OR exp quality of life/ OR exp self-concept/ OR exp spirituality/ OR exp symptom/ OR exp terminal care/ OR exp wellness/	Anger OR “Benefit finding” OR Bereavement OR Body image OR Burden OR Coping OR Cope OR Death OR Dying OR Grief OR Identity OR “Intrusive thoughts” OR “Positive affect” OR “Post-traumatic growth” OR “Post-traumatic stress” OR Psychosocial OR “Quality-of-life” OR Self-appraisal OR Self-concept OR Self-discontent OR Self-esteem OR Self-identity OR “Social support” OR Somatic OR Somatisation OR Wellbeing OR Well-being	accept* OR adjust* OR anxiet* OR attitude* OR belief* OR (cognitive ADJ3 function*) OR depress* OR discriminat* OR distress* OR emotion* OR (emotion* ADJ3 disturb*) OR existential* OR experience* OR fear* OR helpless* OR hope* OR hopeless* OR intima* OR journey* OR lonel* OR marriage* OR (outlook ADJ3 life) OR perception* OR perspective* OR preoccupation* OR (psycholog* ADJ3 distress) OR (psycholog* ADJ3 need*) OR psycho-oncolog* OR (psychosocial* ADJ3 need*) OR (quality ADJ3 life) OR regret* OR relationship* OR (satisfaction ADJ3 care) OR (satisfaction ADJ3 life) OR sexual* OR social* OR (social ADJ3 activit*) OR (social ADJ3 function*) OR (social ADJ3 support*) OR (social ADJ3 well-being) OR spirit* OR stigma* OR survivor* OR (unmet ADJ4 need*) OR worr*

**Table A3:** Medline search terms.

PEO Element	Medline Subject Headings (MeSH)	Synonyms/Free Text Keywords	With Truncation/Wild Card Use
*Population (P):* *People diagnosed with pancreatic cancer*	exp Pancreatic Neoplasms/or (exp Pancreas/and exp Neoplasms/)		Pancrea* adj4 cancer*.m_titl. OR Pancrea* adj4 tumo#r*.m_titl. OR Pancrea* adj4 carcinoma*.m_titl. OR Pancrea* adj4 adenoma*.m_titl. OR Pancrea* adj4 adenocarcinoma*.m_titl. OR Pancrea* adj4 neoplasm*.m_titl.
	exp patients/ OR exp persons/	people.m_titl.	Patient*.m_titl. OR (people adj2 with).m_titl. OR Survivor*.m_titl. OR Person*.m_titl.
*Exposure (E):* *experiences of living (and dying with) pancreatic cancer*	anger/ OR exp anxiety/ OR attitude/ OR attitude to death/ OR attitude to health/ OR attitude to illness/ OR exp bereavement/ OR adaptation, psychological/ OR death/ OR depression/ OR exp emotions/ OR fear/ OR exp grief/ OR exp health/ OR exp life change events/ OR psychological distress/ OR mental health/ OR (neoplasms/AND psychology/) OR psychosocial support systems/ OR quality-adjusted life years/ OR exp quality of life/ OR exp self concept/ OR spirituality/ OR exp symptoms/ OR exp terminally ill/ OR wellness/	Anger OR “Benefit finding” OR Bereavement OR Body image OR Burden OR Coping OR Cope OR Death OR Dying OR Grief OR Identity OR “Intrusive thoughts” OR “Positive affect” OR “Post-traumatic growth” OR “Post-traumatic stress” OR Psychosocial OR “Quality-of-life” OR Self-appraisal OR Self-concept OR Self-discontent OR Self-esteem OR Self-identity OR “Social support” OR Somatic OR Somatisation OR Wellbeing OR Well-being	OR Accept* OR Adjust* OR Anxiet* OR Attitude* OR Belief* OR (cognitive adj3 function*) OR Depress* OR Discriminat* OR Distress* OR Emotion* OR (Emotion* adj3 disturb*) OR Existential* OR Experience* OR Fear* OR Helpless* OR Hope* OR Hopeless* OR Intima* OR Journey* OR Lonel* OR Marriage* OR (Outlook adj3 life) OR Perception* OR Perspective* OR Preoccupation* OR (Psycholog* adj3 distress) OR (Psycholog* adj3 need*) OR Psycho-oncolog* OR (Psychosocial* adj3 need*) OR (Quality adj3 life) OR Regret* OR Relationship* OR (Satisfaction adj3 care) OR (Satisfaction adj3 life) OR Sexual* OR Social* OR (Social adj3 activit*) OR (Social adj3 function*) OR (Social adj3 support*) OR (Social adj3 well-being) OR Spirit* OR Stigma* OR Survivor* OR (unmet adj4 need*) OR Worr*

**Table A4:** PsycINFO search terms.

PEO Element	Thesaurus of Psychological Index Terms	Synonyms/Free Text Keywords	With Truncation/Wild Card Use
*Population (P):* *People diagnosed with pancreatic cancer*	exp pancreatic neoplasms/ OR (exp pancreas/AND exp neoplasms/)		(Pancrea* adj4 tumor*) OR (Pancrea* adj4 carcinoma*) OR (Pancrea* adj4 adenoma*) OR (Pancrea* adj4 adenocarcinoma*) OR (Pancrea* adj4 neoplasm*)
	exp patients/ OR exp persons/	People	“Patient*” OR “(people adj2 with)” OR “Survivor*” OR “Person*”
*Exposure (E):* *experiences of living (and dying with) pancreatic cancer*	exp anger/ OR exp attitude/ OR exp bereavement/ OR exp body image/ OR exp coping behavior/ OR exp emotions/ OR exp death/ OR exp dying/ OR exp grief/ OR exp identity/ OR exp posttraumatic stress disorder/ OR exp psychosocial adjustment/ OR exp quality of life/ OR exp self concept/ OR exp self identity/ OR exp social support/ OR exp well being/	“Benefit finding” OR “Burden” OR “Intrusive thoughts” OR “Positive affect” OR “Post-traumatic growth” OR “Self-appraisal” OR “Self-discontent” OR “Self-esteem” OR “Somatisation” OR “Well-being”	“Accept*” OR “Adjust*” OR “Anxiet*” OR “Belief*” OR “(cognitive adj3 function*)” OR “Depress*” OR “Discriminat*” OR “Distress*” OR”(Emotion* adj3 disturb*)” OR “Existential*” OR “Experience*” OR “Fear*” OR “Helpless*” OR “Hope*” OR “Hopeless*” OR “Intima*” OR “Journey*” OR “Lonel*” OR “Marriage*” OR “(Outlook adj3 life)” OR “Perception*” OR “Perspective*” OR “Preoccupation*” OR “(Psycholog* adj3 distress)” OR “(Psycholog* adj3 need*)” OR “Psycho-oncolog*” OR “(Psychosocial* adj3 need*)” OR “(Quality adj3 life)” OR “Regret*” OR “Relationship*” OR “(Satisfaction adj3 care)” OR “(Satisfaction adj3 life)” OR “Sexual*” OR “Social*” OR “(Social adj3 activit*)” OR “(Social adj3 function*)” OR “(Social adj3 support*)” OR “(Social adj3 well-being)” OR “Spirit*” OR “Stigma*” OR “Survivor*” OR “(unmet adj4 need*)” OR “Worr*”

**Table A5:** Scopus search terms.

PEO Element	Synonyms/Free Text Keywords	With Truncation/Wild Card Use
*Population (P):* *People diagnosed with pancreatic cancer*		(pancrea* W/4 cancer*) OR (pancrea* W/4 tumor*) OR (pancrea* W/4 carcinoma) OR (pancrea* W/4 adenoma*) OR (pancrea* W/4 adenocarcinoma*) OR (pancrea* W/4 neoplasm)
	People	“Patient*” OR “(people W/2 with)” OR “Survivor*” OR “Person*”
*Exposure (E):* *experiences of living (and dying with) pancreatic cancer*	“Anger” OR “Benefit finding” OR “Bereavement” OR “Body image” OR “Burden” OR “Coping” OR “Cope” OR “Death” OR “Dying” OR “Grief” OR “Identity” OR “Intrusive thoughts” OR “Positive affect” OR “Post-traumatic growth” OR “Post-traumatic stress” OR “Psychosocial” OR “Quality-of-life” OR “Self-appraisal” OR “Self-concept” OR “Self-discontent” OR “Self-esteem” OR “Self-identity” OR “Social support” OR “Somatic” OR “Somatisation” OR “Wellbeing” OR “Well-being”	“Accept*” OR “Adjust*” OR “Anxiet*” OR “Attitude*” OR “Belief*” OR “(cognitive W/3 function*)” OR “Depress*” OR “Discriminat*” OR “Distress*” OR “Emotion*” OR “(Emotion* W/3 disturb*)” OR “Existential*” OR “Experience*” OR “Fear*” OR “Helpless*” OR “Hope*” OR “Hopeless*” OR “Intima*” OR “Journey*” OR “Lonel*” OR “Marriage*” OR “(Outlook W/3 life)” OR “Perception*” OR “Perspective*” OR “Preoccupation*” OR “(Psycholog* W/3 distress)” OR “(Psycholog* W/3 need*)” OR “Psycho-oncolog*” OR “(Psychosocial* W/3 need*)” OR “(Quality W/3 life)” OR “Regret*” OR “Relationship*” OR “(Satisfaction W/3 care)” OR “(Satisfaction W/3 life)” OR “Sexual*” OR “Social*” OR “(Social W/3 activit*)” OR “(Social W/3 function*)” OR “(Social W/3 support*)” OR “(Social W/3 well-being)” OR “Spirit*” OR “Stigma*” OR “Survivor*” OR “(unmet W/4 need*)” OR “Worr*”

**Table A6:** Web of Science search terms.

PEO Element	Synonyms/Free Text Keywords	With Truncation/Wild Card Use
*Population (P):* *People diagnosed with pancreatic cancer*		(Pancrea* NEAR/4 cancer*) OR (Pancrea* NEAR/4 tumor*) OR (Pancrea* NEAR/4 carcinoma*) OR (Pancrea* NEAR/4 adenoma*) OR (Pancrea* NEAR/4 adenocarcinoma*) OR (Pancrea* NEAR/4 neoplasm*)
	People	“Patient*” OR (people NEAR/2 with) OR “Survivor*” OR “Person*”
*Exposure (E):* *experiences of living (and dying with) pancreatic cancer*	“Anger” OR “Benefit finding” OR “Bereavement” OR “Body image” OR “Burden” OR “Coping” OR “Cope” OR “Death” OR “Dying” OR “Grief” OR “Identity” OR “Intrusive thoughts” OR “Positive affect” OR “Post-traumatic growth” OR “Post-traumatic stress” OR “Psychosocial” OR “Quality-of-life” OR “Self-appraisal” OR “Self-concept” OR “Self-discontent” OR “Self-esteem” OR “Self-identity” OR “Social support” OR “Somatic” OR “Somatisation” OR “Wellbeing” OR “Well-being”	“Accept*” OR “Adjust*” OR “Anxiet*” OR “Attitude*” OR “Belief*” OR (cognitive NEAR/3 function*) OR “Depress*” OR “Discriminat*” OR “Distress*” OR “Emotion*” OR (Emotion* NEAR/3 disturb*) OR “Existential*” OR “Experience*” OR “Fear*” OR “Helpless*” OR “Hope*” OR “Hopeless*” OR “Intima*” OR “Journey*” OR “Lonel*” OR “Marriage*” OR (Outlook NEAR/3 life) OR “Perception*” OR “Perspective*” OR “Preoccupation*” OR (Psycholog* NEAR/3 distress) OR (Psycholog* NEAR/3 need*) OR “Psycho-oncolog*” OR (Psychosocial* NEAR/3 need*) OR (Quality NEAR/3 life) OR “Regret*” OR “Relationship*” OR (Satisfaction NEAR/3 care) OR (Satisfaction NEAR/3 life) OR “Sexual*” OR “Social*” OR (Social NEAR/3 activit*) OR (Social NEAR/3 function*) OR (Social NEAR/3 support*) OR (Social NEAR/3 well-being) OR “Spirit*” OR “Stigma*” OR “Survivor*” OR (unmet NEAR/4 need*) OR “Worr*”

**Table A7:** World Health Organization Global Index Medicus search terms.

PEO Element	Synonyms/Free Text Keywords	With Truncation/Wild Card Use
*Population (P):* *People diagnosed with pancreatic cancer*	“Pancreatic cancer” OR “Pancreatic tumor” OR “Pancreatic carcinoma” OR “Pancreatic adenoma” OR “Pancreatic adenocarcinoma” OR “Pancreatic neoplasm”	Pancrea* AND (cancer* OR tumo?r* OR carcinoma* OR adenoma* OR adenocarcinoma* OR neoplasm*)
	People	Patient* OR “People with” OR Survivor* OR Person*
*Exposure (E):* *experiences of living (and dying with) pancreatic cancer*	Anger OR “Benefit finding” OR “Bereavement” OR “Body image” OR Burden OR Coping OR Cope OR Death OR Dying OR Grief OR Identity OR “Intrusive thoughts” OR “Positive affect” OR “Post-traumatic growth” OR “Post-traumatic stress” OR “Psychosocial” OR “Quality of life” OR “Life quality” OR “Self-appraisal” OR “Appraisal of self” OR “Self-concept” OR “Concept of self” OR “Self-discontent” OR “Discontent with self” OR “Self-esteem” OR “Esteem of self” OR “Self-identity” OR “Identity of self” OR “Social support” OR “Support, social” OR Somatic OR Somatization OR Wellbeing OR “Well-being”	Accept* OR Adjust* OR Anxiet* OR Attitude* OR Belief* OR “Cognitive function” OR “Function, cognitive” OR Depress* OR Discriminat* OR Distress* OR Emotion* OR “Emotional disturbance” OR “Disturbance, emotional” OR Existential* OR Experience* OR Fear* OR Helpless* OR Hope* OR Hopeless* OR Intimacy OR Journey OR Loneliness OR Marriage OR “Life outlook” OR “Outlook on life” OR Perception* OR Perspective* OR Preoccupation* OR “Psychological distress” OR “Distress, psychological” OR “Psychological need” OR “Need, psychological” OR “Psycho-oncology” OR “Oncology, psycho-“ OR “Psychosocial need” OR “Need, psychosocial” OR “Quality of life” OR “Life quality” OR Regret* OR Relationship* OR “Satisfaction with care” OR “Care satisfaction” OR “Satisfaction with life” OR “Life satisfaction” OR Sexual* OR “Social activity” OR “Activity, social” OR “Social function” OR “Function, social” OR “Social support” OR “Support, social” OR “Social well-being” OR “Well-being, social” OR Spirit* OR Stigma* OR Survivor* OR “Unmet need” OR “Need, unmet” OR Worr*

## Appendix B

Appendix B—Table A8, Table A9, Table A10 and Table A11: coding process for each theme.

**Table A8:** Theme 1: “I felt like the sky was falling”: From ambiguous symptoms to the shock and threat of diagnosis.

Examples of Line-by-Line Codes	Descriptive Category	Interpretation
“I was thinking well maybe it is just something that’s quite benign. You know, a benign growth. So when they did say it’s cancer that really… sledgehammered me…”	Minimising early symptoms → shock of diagnosis	Participants initially normalised symptoms, then experienced profound shock at diagnosis.
“To say you’ve got 3 months, that was pretty rugged when 5 days previously I was as fit as a Mallee bull. Never had a sick day in my life.”	Disruption of ‘healthy identity’	Diagnosis experienced as incompatible with perception of self as a healthy person.
“(S)he said… ‘(Y)ou have pancreatic cancer. It’s fatal. The professor will come and talk to you in a minute. Goodbye.’ Like that. I thought I was going to be run over by a train.” “And suddenly there is such a diagnosis. (…) It almost hit me with a blow. And I was overwhelmed because I couldn’t do anything with it at that moment.”	Shock and disbelief at communication of catastrophic diagnosis/prognosis	Abrupt communication of diagnosis and poor prognosis provoked shock, fear, and sense of existential threat.
“Pancreatic cancer as a whole, statistically, is basically a death sentence. It just depends on how soon or late it’s going to happen.”	Stigma of pancreatic cancer: ‘A death sentence’	Widespread perception of pancreatic cancer as rapidly lethal shaped emotional impact.
“Life is full of shocks. It’s not the same shock as knowing that you’ve got cancer but nevertheless you do get a bit bomb proof as it were to shocks.”	Disbelief	Diagnosis experienced as surreal or unbelievable despite symptoms.

**Table A9:** Theme 2: “If we’d only known we could have got that earlier”: Navigating care, information, and support.

Examples of Line-by-Line Codes	Descriptive Category	Interpretation
“Well, I kind of feel like I didn’t have a choice. That is what I had to do. That is what’s going to happen, and this is what you have to do, so I don’t know if I had a choice. Well, I guess I could’ve chose not to do anything.”	Perceived lack of decision-making autonomy	Initial treatment decisions were often experienced as urgent and predetermined.
“… a lot of information. My husband or my dad was there thankfully because I found that I just couldn’t take it all in”	Information overload	Clinical consultations experienced as overwhelming, limiting ability to absorb and retain information.
“There’s a lot of questions that you think of but you don’t know who to ask because they’re really stupid questions, or you think that they’re stupid…”	System fragmentation	People experienced uncertainty about who to contact for information, advice, and support.
“I remember going with my dad to the GP to find out answers [as to] why my dad had stomach aches … with this GP I had to go with him and say look can my dad have something done or can you forward this on…you have to push it along I think.”	Care partner advocacy	Care partners often acted as advocates to obtain investigations, appointments, and support.
“Doctors inform them but patients are left with questions. They ask me what they should eat and if they will get better and I tell them that they should ask these questions to their doctor.” (Nurse)	Unmet information needs	Information was often perceived as insufficiently practical, personalised, or timely (for example, around diet, prognosis, symptoms).
“The patients searched international websites for information as they did not find leaflets handed out at follow-up relevant or detailed enough.”	Self-directed information seeking	People supplemented professional advice with independent information seeking.

**Table A10:** Theme 3: “Things are not like they used to be…”: Embodied disruption, relational change, and reworking everyday life.

Examples of Line-by-Line Codes	Descriptive Category	Interpretation
“I have no appetite, so I force myself to eat. How can I gain weight for chemotherapy if I don’t eat?”	Eating changes & treatment dependency	Food and eating became tied to survival and continuation of treatment(s).
“Others felt that their medications were a visible and troubling reminder of their illness: ‘I’m reminded of it every meal when I take Creon.’”	Illness reminders	Everyday routines (such as taking PERT) symbolised the permanence of illness and disrupted people’s sense of normality.
“(T)hese symptoms… inhibited their ability to go out for fear of leakage or lack of ready access to bathroom facilities. As we centre our socialisation to a large extent on eating, the experience of these symptoms caused some participants to become so fearful, they became housebound.”	Social withdrawal	Symptoms (particularly gastrointestinal) restricted social life and everyday activities.
“I felt down. I felt emotional, weepy. I felt hopeless and couldn’t do things around the house like I used to do. Uh. Couldn’t do anything really.”	Role disruption & altered identity	Changes in functioning disrupted identity, social roles, and daily life.
“My family gets nervous if I act like I don’t have an appetite, and it makes me feel very stressed.”	Relational strain over symptoms	Symptom management (e.g., appetite, weight) became a source of relational tension.
“You are never, ever, ever the same, regardless, even if it’s a positive outcome, and that’s not bad, but you are never, ever, ever the same.”	New normal	Participants described adapting to changed sense of self and everyday life.

**Table A11:** Theme 4: “Living on borrowed time”: Coping with uncertainty and mortality.

Examples of Line-by-Line Codes	Descriptive Category	Interpretation
“Waiting for the results, that’s the hardest part… at that point, I couldn’t eat… My stomach was churning like a milk truck. I almost got sick right there in the room.” “It’s just a wonderful feeling that I want to cry. I want to cry every time. I made it through another scan. Nothing showed up. I feel healthy. I don’t feel like a weakling anymore.”	Fears of recurrence and progression manifesting in ‘scanxiety’	Participants described recurring cycles of anticipatory anxiety, physiological distress, reassurance, and renewed uncertainty centred on fears of recurrence and disease progression, followed by relief and reassurance of “good news”.
“I just never plan anything big because I don’t know if I’ll be dead or if I’ll be in treatment.” “…I’m just living on borrowed time…Any time I plan on something that’s going to take more than a year. I just don’t—I just put it out of my mind, because I don’t feel like I’m going to be around that much longer…I can’t really plan on a future.”	Impact of existential awareness and uncertainty on planning and foreshortened future	Awareness of mortality and uncertainty about prognosis disrupted future planning and made it difficult for participants to imagine long-term goals, roles, or life events.
“I like to be realistic and confront things head-on, so… balance being a realist with kicking myself in the butt to keep going. I have to remind myself, you’re not dead yet!” “I want to grab what I can now. Hopefully these pains will get a bit better and I can do a bit more. I really felt quite optimistic last week; in fact, I suppose I’m angry because I was quite high on Monday and yesterday I was quite low. Yesterday I suddenly thought I’m not going to get the drug. I’m going to die soon. Monday, I was going to live…”	Balancing fatalism and optimism, hope versus fear	In the face of unpredictable disease course and uncertainty, participants continually recalibrated between hope and fear, striving to remain realistic about prognosis while maintaining motivation to continue living.
“I know there is no cure, and it’s interesting seeing a palliative care physician, which I do also; she says at some stage you’ve really got to pull the plug on treatment and I totally agree with her. And get on and enjoy the rest of your life while you can. If after two months I’m still in no pain and my CT scan is fine, and everything, I might go back on another course of chemo. That’s my plan.” “It’s a lifelong journey. I think about this often. It’s not a … oh, I beat it. You don’t beat it. You work at it. It’s a job and you gotta keep working at it and make sure that you do everything humanly possible to try and overcome this. Not overcome it but to live with it.”	Coping with uncertainty and awareness of mortality	Rather than resolving uncertainty, participants described ongoing efforts to live meaningfully alongside awareness of mortality and the possibility of disease progression, cancer recurrence, or death.
