# Supplementary material for: Negotiating Mortality: A Qualitative Systematic Review and Thematic Synthesis of the Psychosocial Experiences of Pancreatic Cancer Across the Illness Trajectory
Source: Cancers (Basel). 2026 Oct 1;18(19):3177. doi: 10.3390/cancers18193177 (PMC13664414; doi:10.3390/cancers18193177)
Supplement: Supplementary file 1 [file cancers-18-03177-s001.zip › Cook et al. 2026 Tables S2-S9.pdf]

- Table S2. Search history and results.
- Table S3. CINAHL Complete (EBSCOhost) search strategy.
- Table S4. Embase (Ovid) search strategy.
- Table S5. MEDLINE (Ovid) search strategy.
- Table S6. APA PsycINFO (Ovid) search strategy.
- Table S7. Scopus search strategy.
- Table S8. Web of Science Core Collection search strategy.
- Table S9. WHO Global Index Medicus search strategy.

Table S2. Search history and results.

Database	Search #1	Update #2	Update #3	No. Records Retrieved*		
				#1	#2	#3
**CINAHL**	21 Aug 2024	28 Feb 2025	4 Sep 2025	544	32	40
**Embase**	27 Aug 2024	28 Feb 2025	4 Sep 2025	1201	103	67
**MEDLINE**	21 Aug 2024	28 Feb 2025	4 Sep 2025	3012	191	148
**PsycINFO**	27 Aug 2024	28 Feb 2025	4 Sep 2025	51	0	0
**Scopus**	28 Aug 2024	28 Feb 2025	4 Sep 2025	634	24	47
**Web of Science**	28 Aug 2024	28 Feb 2025	4 Sep 2025	953	80	43
**WHO GIM**	28 Aug 2024	28 Feb 2025	4 Sep 2025	34	0	0

**Includes duplicates*

The same search strategy was rerun for update searches. Date restrictions were applied to identify records added since the preceding search date and minimise duplicate retrievals. The most recent searches are reflected in the search strategies below, with the exception of ***Table S8*** which provides the entire search string and total results at initial search, since this database does not provide query by query results in 'advanced search'.

Note: Searches were all conducted on 4 September 2025, but using the date limits on databases at this time provided various dates in the search strings below.

Table S3. CINAHL Complete (EBSCOhost) search strategy.

**CINAHL Complete (01/02/2025–30.09.2025)**		
**Search line #**	**Query**	**No. results**
S1	TI (((MH "Pancreatic Neoplasms+") OR ((MH "Pancreas+") AND (MH "Neoplasms+")))	15917
S2	TI (pancrea* N5 (cancer* OR tumo?r* OR carcinoma* OR adenoma* OR adenocarcinoma* OR neoplasm*)))	13423
S3	TI ((MH "Patients") OR (MH "Cancer Patients") OR (MH "Hospice Patients"))	5901
S4	TI People	112048
S5	TI (Patient* OR (people W2 with) OR survivor* OR person*)	1179228
S6	S1 OR S2	20666
S7	S3 OR S4 OR S5	1216872
S8	(MH "Anger") OR (MH "Anxiety+") OR (MH "Attitude") OR (MH "Attitude to Death+") OR (MH "Attitude to Health") OR (MH "Attitude to Illness") OR (MH "Bereavement+") OR (MH "Coping") OR (MH "Death") OR (MH "Depression") OR (MH "Emotions+") OR (MH "Fear") OR (MH "Grief+") OR (MH "Health+") OR (MH "Life Experiences") OR (MH "Life Change Events+") OR (MH "Psychological Distress") OR (MH "Psychological Well-Being") OR (MH "Psycho-Oncology") OR (MH "Psychosocial Intervention") OR (MH "Quality-Adjusted Life Years") OR (MH "Quality of Life") OR (MH "Self Concept+") OR (MH "Spiritual Distress (NANDA)") OR (MH "Spiritual Distress (Saba CCC)") OR (MH "Support, Psychosocial") OR (MH "Symptom Burden") OR (MH "Symptom Distress") OR (MH "Terminally Ill Patients+") OR (MH "Wellness")	1267797

S9	TI (Anger OR "Benefit finding" OR Bereavement OR Body image OR Burden OR Coping OR Cope OR Death OR Dying OR Grief OR Identity OR "Intrusive thoughts" OR "Positive affect" OR "Post-traumatic growth" OR "Post-traumatic stress" OR Psychosocial OR "Quality-of-life" OR Self-appraisal OR Self-concept OR Self-discontent OR Self-esteem OR Self-identity OR "Social support" OR Somatic OR Somatisation OR Wellbeing OR Well-being)	252008
S10	TI (OR Accept* OR Adjust* OR Anxiet* OR Attitude* OR Belief* OR (cognitive N3 function*) OR Depress* OR Discriminat* OR Distress* OR Emotion* OR (Emotion* N3 disturb*) OR Existential* OR Experience* OR Fear* OR Helpless* OR Hope* OR Hopeless* OR Intima* OR Journey* OR Lonel* OR Marriage* OR (Outlook N3 life) OR Perception* OR Perspective* OR Preoccupation* OR (Psycholog* N3 distress) OR (Psycholog* N3 need*) OR Psycho-oncolog* OR (Psychosocial* N3 need*) OR (Quality N3 life) OR Regret* OR Relationship* OR (Satisfaction N3 care) OR (Satisfaction N3 life) OR Sexual* OR Social* OR (Social N3 activit*) OR (Social N3 function*) OR (Social N3 support*) OR (Social N3 well-being) OR Spirit* OR Stigma* OR Survivor* OR (unmet N4 need*) OR Worr*)	942774
S11	S6 AND S7	4220
S12	S8 OR S9 OR S10	1916202
S13	S11 AND S12 (Search options: 01/02/2025–30/09/2025)	40

Table S4. Embase (Ovid) search strategy.

**Embase <1974 to 2025 September 02>**		
**Search line #**	**Query**	**No. results**
1	exp pancreatic neoplasm/	225829
2	exp pancreas/ and exp neoplasm/	44047
3	((pancrea* adj4 cancer*) or (pancrea* adj4 tumor*) or (pancrea* adj4 carcinoma*) or (pancrea* adj4 adenoma*) or (pancrea* adj4 adenocarcinoma*) or (pancrea* adj4 neoplasm*)).m_titl.	111322
4	1 or 2 or 3	248983
5	'exp patients/ or exp persons/ or exp cancer patient/ or exp hospice patient/	4337943
6	people.m_titl.	173534
7	(patient* or (people adj2 with) or survivor* or person*).m_titl.	4243306
8	5 or 6 or 7	7372563
9	4 and 8	73948
10	exp anger/ or exp anxiety/ or exp attitude/ or exp attitude to death/ or exp attitude to health/ or exp attitude to illness/ or exp bereavement/ or exp psychological adaptation/ or exp death/ or exp depression/ or exp emotions/ or exp fear/ or exp grief/ or exp health/ or exp life change event/ or exp psychological distress/ or exp mental health/ or (exp neoplasms/ and exp psychology/) or exp psychosocial care/ or exp quality-adjusted life year/ or exp quality of life/ or exp self-concept/ or exp spirituality/ or exp symptom/ or exp terminal care/ or exp wellness/	6202260
11	(Anger or "Benefit finding" or Bereavement or Body image or Burden or Coping or Cope or Death or Dying or Grief or Identity or "Intrusive thoughts" or "Positive affect" or "Post-traumatic growth" or "Post-traumatic stress" or Psychosocial or "Quality-of-life" or Self-appraisal or Self-concept or Self-discontent or Self-esteem or Self-identity or "Social support" or Somatic or Somatisation or Wellbeing or Well-being).m_titl.	622859
12	(accept* or adjust* or anxiet* or attitude* or belief* or (cognitive adj3 function*) or depress* or discriminat* or distress* or emotion* or (emotion* adj3 disturb*) or existential* or experience* or fear* or helpless* or hope* or hopeless* or intima* or journey* or lonel* or marriage* or (outlook adj3 life) or perception* or perspective* or preoccupation* or (psycholog* adj3 distress) or (psycholog* adj3 need*) or psycho-oncolog* or (psychosocial* adj3 need*) or (quality adj3 life) or regret* or relationship* or (satisfaction adj3 care) or (satisfaction adj3 life) or sexual* or social* or (social adj3 activit*) or (social adj3 function*) or (social adj3 support*) or (social adj3 well-being) or spirit* or stigma* or survivor* or (unmet adj4 need*) or worr*).m_titl.	2459396
13	10 or 11 or 12	7671847
14	9 and 13	18797

15	(pancreatic neoplasm or (pancreas and neoplasm) or ((pancrea* adj4 cancer*) or (pancrea* adj4 tumor*) or (pancrea* adj4 carcinoma*) or (pancrea* adj4 adenoma*) or (pancrea* adj4 adenocarcinoma*) or (pancrea* adj4 neoplasm*))).m_titl.	111421
16	('exp patients or persons or cancer patient or hospice patient or people or (patient* or (people adj2 with) or survivor* or person*)).m_titl.	4321207
17	15 and 16	18377
18	(anger or anxiety or attitude or attitude to death or attitude to health or attitude to illness or bereavement or psychological adaptation or death or depression or emotions or fear or grief or health or life change event or psychological distress or mental health or (neoplasms and psychology) or psychosocial care or quality-adjusted life year or quality of life or self-concept or spirituality or symptom or terminal care or wellness or (Anger or "Benefit finding" or Bereavement or Body image or Burden or Coping or Cope or Death or Dying or Grief or Identity or "Intrusive thoughts" or "Positive affect" or "Post-traumatic growth" or "Post-traumatic stress" or Psychosocial or "Quality-of-life" or Self-appraisal or Self-concept or Self-discontent or Self-esteem or Self-identity or "Social support" or Somatic or Somatisation or Wellbeing or Well-being) or (accept* or adjust* or anxiet* or attitude* or belief* or (cognitive adj3 function*) or depress* or discriminat* or distress* or emotion* or (emotion* adj3 disturb*) or existential* or experience* or fear* or helpless* or hope* or hopeless* or intima* or journey* or lonel* or marriage* or (outlook adj3 life) or perception* or perspective* or preoccupation* or (psycholog* adj3 distress) or (psycholog* adj3 need*) or psycho-oncolog* or (psychosocial* adj3 need*) or (quality adj3 life) or regret* or relationship* or (satisfaction adj3 care) or (satisfaction adj3 life) or sexual* or social* or (social adj3 activit*) or (social adj3 function*) or (social adj3 support*) or (social adj3 well-being) or spirit* or stigma* or survivor* or (unmet adj4 need*) or worr*)).m_titl.	3663670
19	17 and 18	1345
20	limit 19 to yr="2025 -Current"	67

Table S5. MEDLINE (Ovid) search strategy.

**Ovid MEDLINE(R) ALL <1946 to September 03, 2025>**		
**Search line #**	**Query**	**No. results**
1	exp Pancreatic Neoplasms/ or (exp Pancreas/ and exp Neoplasms/)	108856
2	(Pancrea* adj4 cancer*).m_titl.	40752
3	(Pancrea* adj4 tumo#r*).m_titl.	1385
4	(Pancrea* adj4 carcinoma*).m_titl.	6963
5	(Pancrea* adj4 adenoma*).m_titl.	431
6	(Pancrea* adj4 adenocarcinoma*).m_titl.	12821
7	(Pancrea* adj4 neoplasm*).m_titl.	3919
8	exp patients/	89610
9	exp Persons/	11790365
10	people.m_titl.	134279
11	(Patient* or (people adj2 with) or Survivor* or Person*).m_titl.	2830319
12	anger/ or exp anxiety/ or attitude/ or attitude to death/ or attitude to health/ or attitude to illness/ or exp bereavement/ or adaptation, psychological/ or death/ or depression/ or exp emotions/ or fear/ or exp grief/ or exp health/ or exp life change events/ or psychological distress/ or mental health/ or (neoplasms/ and psychology/) or psychosocial support systems/ or quality-adjusted life years/ or exp quality of life/ or exp self concept/ or spirituality/ or exp symptoms/ or exp terminally ill/ or wellness/	1490673
13	(Anger or "Benefit finding" or Bereavement or Body image or Burden or Coping or Cope or Death or Dying or Grief or Identity or "Intrusive thoughts" or "Positive affect" or "Post-traumatic growth" or "Post-traumatic stress" or Psychosocial or "Quality-of-life" or Self-appraisal or Self-concept or Self-discontent or Self-esteem or Self-identity or "Social support" or Somatic or Somatisation or Wellbeing or Well-being).m_titl.	491276
14	(Accept* or Adjust* or Anxiet* or Attitude* or Belief* or (cognitive adj3 function*) or Depress* or Discriminat* or Distress* or Emotion* or (Emotion* adj3 disturb*) or Existential* or Experience* or Fear* or Helpless* or Hope* or Hopeless* or Intima* or Journey* or Lonel* or Marriage* or (Outlook adj3 life) or Perception* or Perspective* or Preoccupation* or (Psycholog* adj3 distress) or (Psycholog* adj3 need*) or Psycho-oncolog* or (Psychosocial* adj3 need*) or (Quality adj3 life) or Regret* or Relationship* or (Satisfaction adj3 care) or (Satisfaction adj3 life) or Sexual* or Social* or (Social adj3	2019333

	activit*) or (Social adj3 function*) or (Social adj3 support*) or (Social adj3 well-being) or Spirit* or Stigma* or Survivor* or (unmet adj4 need*) or Worr*).m_titl.	
15	1 or 2 or 3 or 4 or 5 or 6 or 7	121757
16	8 or 9 or 10 or 11	12968678
17	12 or 13 or 14	3251461
18	15 and 16 and 17	3222
19	limit 18 to yr="2025 -Current"	148

Table S6. APA PsycINFO (Ovid) search strategy.

**APA PsycInfo <1806 to August 2025 Week 4>**		
**Search line #**	**Query**	**No. results**
1	((Pancrea* adj4 tumor*) or (Pancrea* adj4 carcinoma*) or (Pancrea* adj4 adenoma*) or (Pancrea* adj4 adenocarcinoma*)).mp. or (Pancrea* adj4 neoplasm*).m_titl. [mp=title, abstract, heading word, table of contents, key concepts, original title, tests & measures, mesh word]	172
2	exp pancreatic neoplasms/ or (exp pancreas/ and exp neoplasms/)	241
3	1 or 2	336
4	exp patients/ or exp persons/	113093
5	people.m_titl.	64416
6	("Patient*" or "(people adj2 with)" or "Survivor*" or "Person*").m_titl.	377727
7	4 or 5 or 6	504200
8	3 and 7	105
9	exp anger/ or exp attitude/ or exp bereavement/ or exp body image/ or exp coping behavior/ or exp emotions/ or exp death/ or exp dying/ or exp grief/ or exp identity/ or exp posttraumatic stress disorder/ or exp psychosocial adjustment/ or exp quality of life/ or exp self concept/ or exp self identity/ or exp social support/ or exp well being/	775557
10	("Benefit finding" or "Burden" or "Intrusive thoughts" or "Positive affect" or "Post-traumatic growth" or "Self-appraisal" or "Self-discontent" or "Self-esteem" or "Somatisation" or "Well-being").m_titl.	55869
11	("Accept*" or "Adjust*" or "Anxiet*" or "Belief*" or "(cognitive adj3 function*)" or "Depress*" or "Discriminat*" or "Distress*" or "(Emotion* adj3 disturb*)" or "Existential*" or "Experience*" or "Fear*" or "Helpless*" or "Hope*" or "Hopeless*" or "Intima*" or "Journey*" or "Lonel*" or "Marriage*" or "(Outlook adj3 life)" or "Perception*" or "Perspective*" or "Preoccupation*" or "(Psycholog* adj3 distress)" or "(Psycholog* adj3 need*)" or "Psycho-oncolog*" or "(Psychosocial* adj3 need*)" or "(Quality adj3 life)" or "Regret*" or "Relationship*" or "(Satisfaction adj3 care)" or "(Satisfaction adj3 life)" or "Sexual*" or "Social*" or "(Social adj3 activit*)" or "(Social adj3 function*)" or "(Social adj3 support*)" or "(Social adj3 well-being)" or "Spirit*" or "Stigma*" or "Survivor*" or "(unmet adj4 need*)" or "Worr*").m_titl.	1156617
12	9 or 10 or 11	1660803
13	8 and 12	53
14	limit 13 to yr="2025 -Current"	0

Table S7. Scopus search strategy.

**Scopus**		
**Search line #**	**Query**	**No. results**
1	((TITLE ((pancrea* W/4 cancer*) OR (pancrea* W/4 tumor*) OR (pancrea* W/4 carcinoma) OR (pancrea* W/4 adenoma*) OR (pancrea* W/4 adenocarcinoma*) OR (pancrea* W/4 neoplasm))) AND (TITLE (people OR "Patient*" OR "(people W/2 with)" OR "Survivor*" OR "Person*"))) AND ((TITLE ("Accept*" OR "Adjust*" OR "Anxiet*" OR "Attitude*" OR "Belief*" OR "(cognitive W/3 function*)" OR "Depress*" OR "Discriminat*" OR "Distress*" OR "Emotion*" OR "(Emotion* W/3 disturb*)" OR "Existential*" OR "Experience*" OR "Fear*" OR "Helpless*" OR "Hope*" OR "Hopeless*" OR "Intima*" OR "Journey*" OR "Lonel*" OR "Marriage*" OR "(Outlook W/3 life)" OR "Perception*" OR "Perspective*" OR "Preoccupation*" OR "(Psycholog* W/3 distress)" OR "(Psycholog* W/3 need*)" OR "Psycho-oncolog*" OR "(Psychosocial* W/3 need*)" OR "(Quality W/3 life)" OR "Regret*" OR "Relationship*" OR "(Satisfaction W/3 care)" OR "(Satisfaction W/3 life)" OR "Sexual*" OR "Social*" OR "(Social W/3 activit*)" OR "(Social W/3 function*)" OR "(Social W/3 support*)" OR	47

	"(Social W/3 well-being)" OR "Spirit*" OR "Stigma*" OR "Survivor*" OR "(unmet W/4 need*)" OR "Worr*")) OR (TITLE ("Anger" OR "Benefit finding" OR "Bereavement" OR "Body image" OR "Burden" OR "Coping" OR "Cope" OR "Death" OR "Dying" OR "Grief" OR "Identity" OR "Intrusive thoughts" OR "Positive affect" OR "Post-traumatic growth" OR "Post-traumatic stress" OR "Psychosocial" OR "Quality-of-life" OR "Self-appraisal" OR "Self-concept" OR "Self-discontent" OR "Self-esteem" OR "Self-identity" OR "Social support" OR "Somatic" OR "Somatisation" OR "Wellbeing" OR "Well-being"))) AND PUBYEAR = 2025	

Table S8. Web of Science Core Collection search strategy.

**Web of Science**		
**Search line #**	**Query**	**No. results**
1	TI=((Pancrea* NEAR/4 cancer*) OR (Pancrea* NEAR/4 tumor*) OR (Pancrea* NEAR/4 carcinoma*) OR (Pancrea* NEAR/4 adenoma*) OR (Pancrea* NEAR/4 adenocarcinoma*) OR (Pancrea* NEAR/4 neoplasm*))	99715
2	(TI=(people)) OR TI=("Patient*" OR (people NEAR/2 with) OR "Survivor*" OR "Person*")	4050478
3	(TI=("Anger" OR "Benefit finding" OR "Bereavement" OR "Body image" OR "Burden" OR "Coping" OR "Cope" OR "Death" OR "Dying" OR "Grief" OR "Identity" OR "Intrusive thoughts" OR "Positive affect" OR "Post-traumatic growth" OR "Post-traumatic stress" OR "Psychosocial" OR "Quality-of-life" OR "Self-appraisal" OR "Self-concept" OR "Self-discontent" OR "Self-esteem" OR "Self-identity" OR "Social support" OR "Somatic" OR "Somatisation" OR "Wellbeing" OR "Well-being")) OR TI=("Accept*" OR "Adjust*" OR "Anxiet*" OR "Attitude*" OR "Belief*" OR (cognitive NEAR/3 function*) OR "Depress*" OR "Discriminat*" OR "Distress*" OR "Emotion*" OR (Emotion* NEAR/3 disturb*) OR "Existential*" OR "Experience*" OR "Fear*" OR "Helpless*" OR "Hope*" OR "Hopeless*" OR "Intima*" OR "Journey*" OR "Lonel*" OR "Marriage*" OR (Outlook NEAR/3 life) OR "Perception*" OR "Perspective*" OR "Preoccupation*" OR (Psycholog* NEAR/3 distress) OR (Psycholog* NEAR/3 need*) OR "Psycho-oncolog*" OR (Psychosocial* NEAR/3 need*) OR (Quality NEAR/3 life) OR "Regret*" OR "Relationship*" OR (Satisfaction NEAR/3 care) OR (Satisfaction NEAR/3 life) OR "Sexual*" OR "Social*" OR (Social NEAR/3 activit*) OR (Social NEAR/3 function*) OR (Social NEAR/3 support*) OR (Social NEAR/3 well-being) OR "Spirit*" OR "Stigma*" OR "Survivor*" OR (unmet NEAR/4 need*) OR "Worr*")	4758799
4	#1 AND #2 AND #3<br>[Timespan: 2025-02-28 to 2025-09-04 (Index Date) (in 'Edit Query #4')]	43

Table S9. WHO Global Index Medicus search strategy.

**WHO Global Index Medicus advanced search, entire search string, no limiter for year**		
**Search line #**	**Query**	**No. results**
1	(ti:(Pancrea* AND (cancer* OR tumo?r* OR carcinoma* OR adenoma* OR adenocarcinoma* OR neoplasm*))) AND (ti:(people OR Patient* OR "People with" OR Survivor* OR Person*)) AND (ti:(Anger OR "Benefit finding" OR "Bereavement" OR "Body image" OR Burden OR Coping OR Cope OR Death OR Dying OR Grief OR Identity OR "Intrusive thoughts" OR "Positive affect" OR "Post-traumatic growth" OR "Post-traumatic stress" OR "Psychosocial" OR "Quality of life" OR "Life quality" OR "Self-appraisal" OR "Appraisal of self" OR "Self-concept" OR "Concept of self" OR "Self-discontent" OR "Discontent with self" OR "Self-esteem" OR "Esteem of self" OR "Self-identity" OR "Identity of self" OR "Social support" OR "Support, social" OR Somatic OR Somatization OR Wellbeing OR "Well-being" OR Accept* OR Adjust* OR Anxiet* OR Attitude* OR Belief* OR "Cognitive function" OR "Function, cognitive" OR Depress* OR Discriminat* OR Distress* OR Emotion* OR "Emotional disturbance" OR "Disturbance, emotional" OR Existential* OR Experience* OR Fear* OR Helpless* OR Hope* OR Hopeless* OR Intimacy OR Journey OR Loneliness OR Marriage OR "Life outlook" OR "Outlook on life" OR Perception* OR Perspective* OR Preoccupation* OR "Psychological distress" OR "Distress, psychological" OR "Psychological need" OR "Need, psychological" OR "Psycho-oncology" OR "Oncology, psycho-" OR "Psychosocial need" OR "Need, psychosocial" OR "Quality of life" OR "Life quality" OR Regret* OR Relationship* OR "Satisfaction with care" OR "Care satisfaction" OR "Satisfaction with life" OR "Life satisfaction" OR Sexual* OR "Social activity" OR "Activity, social" OR "Social function" OR "Function, social" OR "Social support" OR	34

	"Support, social" OR "Social well-being" OR "Well-being, social" OR Spirit* OR Stigma* OR Survivor* OR "Unmet need" OR "Need, unmet" OR Worr*))	
